# Supplementary material for: Childhood abuse and perinatal outcomes for mother and child: A systematic review of the literature
Source: PLoS One. 2024 May 24;19(5):e0302354. doi: 10.1371/journal.pone.0302354 (PMC11125509; doi:10.1371/journal.pone.0302354)
Supplement: S1 Appendix — (DOCX) [file pone.0302354.s001.docx]

# S1 Appendix: Data Extraction Sheet for Quality Assessment and Risk of Bias

Notes on using a data extraction form to extract data from included studies, including assessing the risk of bias:

- Complete one form for each article.
- Be consistent in the order and style you use to describe the information.
- Highlight any missing information as unclear or not described, to make it clear that the information was not included in the description of the study, not that you forgot to extract it.
- It may be reasonable to make assumptions about how the study was conducted, but these assumptions must be reported. Supplement ambiguous quotes with a decision of ‘Probably done’ or ‘Probably not done’, providing a rationale for the assumption.

You must try to contact authors for any additional information or clarification required.

**Study ID:**

**Name of reviewer**:

# Methods

**Aims / hypotheses:**

## Participant recruitment

**Is this part of a larger cohort study (if yes pls state name):**

**Eligibly / exclusion criteria:**

**Methods of recruitment of participants**:

**Geographic location/ setting:**

**Participant Recruitment**

**Eligible for inclusion**:

**Excluded as not eligible**:

**Refused to participate/ attrition / other reason:**

**Final sample size:**

**Number of groups** (including control groups);

**Were non-participants compared to participants**?

**Please rate the selection procedures** –

- *strong when a sample was very likely to be representative of the target population and the study had a participation rate over 80%,*
- *moderate when a sample was somewhat likely to be representative of the target population and had a 60–79% participation rate,*
- *weak when there was a different response rate or it was not stated*

**Participant Characteristics**

**Age (M, SD):**

**Are participants described in detail (e.g., education, employment, income): yes /no**

**Parity: nulliparous/ multiparous/ mixed / retrospective (i.e. not currently pregnant)**

**Materials**

**Timing of assessment(s):**

**Instruments used and construct assessed** (e.g., depression (CESD) *please list***):**

**Type of abuse examined:**

**How defined?** (e.g., age limit)

Interview/ self-report

**Outcome measures** *(please list):*

**Please rate the data collection methods** –

- *A strong rating is given for studies that used tools that were both valid and reliable.*
- *A moderate rating is given when the study included validity data.*
- *A weak rating was given when there was no demonstration of evidence for the validity and reliability of the measure used in the study or if only reliability was described and no information about validity given*.

**Design**

**Describe the design:**

**Describe main analysis:**

**Covariates controlled?** *If yes please state***.**

**Please rate the study design** -

- *strong rating when the design was longitudinal, included control group or robust analyses that controlled for all possible confounders;*
- *a moderate rating was assigned to cross sectional, correlational design or minimal confounders controlled,*
- *a weak rating for all other designs or studies where designs were not stated and no confounders were controlled for.*

**Please rate the statistical analysis** –

- *A strong rating for robust analyses that are appropriate to answer the hypotheses/aims (effect sizes and CIs reported with assumptions checked)*
- *A medium rating for analyses that are less robust. This would include correlational or descriptive studies or studies that do not report prior assumptions, effect sizes or CIs.*
- *A weak rating for analyses that are not well described or inappropriate for the hypotheses*,

**Is the study suitable for meta-analysis?** Yes/no

*To be suitable study needs to report means and SD/SE for treatment and control groups.*

# Results

**Outcomes** *(list briefly, if they are lengthy state ‘refer to article for further detail’).*

**Further information required from author?**

*If yes,* **Author contact details for study:**

Correspondence with authors successful or not;

What information was received and when

Will any additional unpublished data supplied be included in the review?

# Risk of Bias

Quality and risk of bias are assessed for the following areas:

1. Selection bias -
2. Detection bias (problems with measurement) -
3. Confounding -
4. Missing information-

Please provide an overall rating for each area based on the total score (strong rating = 3, Medium = 2, weak = 1). Use the following as a guide (and your own judgement):

- Low risk of bias (high ratings for all areas) = 12
- Low-med risk of bias = 8.5-11.5
- Med risk of bias, med ratings for all areas = 8
- Med-high risk of bias =4. 5-7.5
- High risk of bias, low rating for all areas = 4
