# Supplementary material for: Childhood abuse and perinatal outcomes for mother and child: A systematic review of the literature
Source: PLoS One. 2024 May 24;19(5):e0302354. doi: 10.1371/journal.pone.0302354 (PMC11125509; doi:10.1371/journal.pone.0302354)
Supplement: S1 Table — (DOCX) [file pone.0302354.s002.docx]

**Supplementary Table 1**

*Assessment of the studies for inclusion in the meta-analysis*

*Criteria*

- *> 2 studies examining the outcome*
- *Comparable samples (while some country variation is acceptable, clinical samples cannot be compared to community samples [e.g., depression sample])*
- *Predictors and outcome variables (the measure does not need to be the same, but the variable assessed does need to be the same)*
- *Comparable timing (important to consider the timing as it can impact the outcome)*
- *Statistical analyses (some outcomes can be standardized, but not all comparable)*

| **Study (first author, year)** | **Samples** | **Comparable** | **Abuse measures** | **Comparable** | **Outcome measures** | **Comparable** | **Timing** | **Comparable** | **Effect size** | **Comments** |
| --- | --- | --- | --- | --- | --- | --- | --- | --- | --- | --- |
| **Antenatal depression** | | | | | | | | | | |
| **Predictors - CPA, CSA or CEA** | | | | | | | | | | |
| ~~Barrios (2015)~~ | ~~Comm, M = 28.0 (6.2), Peru~~ | ~~Yes for age & comm sample~~ | ~~CPA & CSA (ACES), ≤ 18 years~~ | ~~Age limit differs~~ | ~~Dep (PHQ-9)~~ | ~~Yes~~ | ~~Early preg.~~ | ~~Yes~~ | ~~aOR~~ | ~~Controls for SES – leave only 2 studies that can be compared~~ |
| ~~Chung (2008)~~ | ~~Comm, M = 24.0 (6.0), US~~ | ~~Yes for age & comm sample~~ | ~~CPA, CSA & CEA (ACES) ≤ 16 yrs~~ | ~~Different age limit, would need to drop CEA~~ | ~~Dep (CESD)~~ | ~~Yes~~ | ~~Early-preg~~ | ~~Yes~~ | ~~aOR~~ | ~~Controls for PICs not comparable~~ |
| ~~Rich-Edwards (2011)~~ | ~~ACCESS & Viva studies, aged 14 – 40+ (Viva, 86.1% aged 25-40, ACCESS 75.3% aged 20-35) US~~ | ~~Yes for age & comm sample~~ | ~~CPA & CSA (PSQ) ≤ 17 yrs~~ | ~~Different age limit~~ | ~~Dep (EPDS)~~ | ~~Yes~~ | ~~Mid-preg.~~ | ~~No~~ | ~~aOR~~ | ~~Only has CPA&CSA overall not comparable~~ |
| ~~Zhong (2016)~~ | ~~Comm, M = 28.1 (6.3), Peru~~ | ~~Yes for age & comm sample~~ | ~~CPA & CSA (CPSAQ)~~ | ~~Yes on age limit, measure differs~~ | ~~Dep (PHQ-9)~~ | ~~Yes~~ | ~~9.2 weeks gest.~~ | ~~yes~~ | ~~aOR~~ |  |
| **Child abuse potential** | | | | | | | | | | |
| ~~Appleyard (2011)~~ |  |  | ~~CPA, CSA (CTS)~~ |  | ~~Offspring victimization (CPS)~~ | ~~No~~ |  |  |  | ~~Outcomes & research questions differ~~ |
| ~~Bert (2009)~~ |  |  | ~~CPA, CSA & CEA (CTQ)~~ |  | ~~Child abuse potential~~ | ~~No~~ |  |  |  |  |
| ~~Choi (2018)~~ |  |  | ~~CPA, CSA & CEA (CTQ) ≤18 yrs~~ |  | ~~Child exposure to harm~~ | ~~No~~ |  |  |  |  |
| **Cortisol** | | | | | | | | | | |
| ~~Bublitz (2012)~~ | ~~BAMBI, M = 26.46 (5.49)~~ |  | ~~ACES (CSA & CPA)~~ |  | ~~CAR (Saliva), wakeup, 30 mins after wake & bedtime~~ |  | ~~2~~^~~nd~~^ ~~& 3~~^~~rd~~^ ~~trimester~~ |  | ~~F~~ | ~~Different analyses~~ |
| ~~Bublitz (2014)~~ | ~~As above~~ | ~~No (same cohort as above)~~ | ~~ACES (CSA and CPA)~~ |  | ~~as above~~ |  | ~~As above~~ |  | ~~B~~ | ~~Leaves only 2 studies that can be included~~ |
| ~~Stephens (2021)~~ | ~~MOMS & Pittsburg PA study, 30.1 (5.1)~~ | ~~Yes~~ | ~~CTQ (CPA, CSA, CEA)~~ |  | ~~CAR (saliva) 30, 60 mins after wake, afternoon & bedtime~~ | ~~Yes for 30 & bedtime~~ | ~~2~~^~~nd~~^ ~~trimester~~ | ~~yes~~ | ~~B~~ |  |
| **Fear of Childbirth** | | | | | | | | | | |
| ~~Heimstad (2006)~~ | ~~Comm, 29.6, Norway~~ |  | ~~CPA & CSA sgl qs~~ |  | ~~Fear of Childbirth (WDEQ)~~ |  | *~~Around 18 weeks gest.~~* |  | *~~t-tests~~* |  |
| ~~Lukasse (2011)~~ | ~~MoBA Norway~~ |  | ~~CA (Norvold)~~ | ~~No~~ | ~~Fear of Childbirth (sgl qs from MoBa questionnaire)~~ | ~~No - uses sgl question (I am really dreading giving birth)~~ | ~~18 & 30 wks gest.~~ |  | ~~LR~~ | ~~Mot comparable on abuse measure and outcome, leave only 2 studies~~ |
| ~~Lukasse (2010a)~~ | ~~BIDENS, 78.7% aged 25-35~~ | ~~Yes age, no culture~~ | ~~CA (CPA, CSA, CEA [Norvold]) ≤ 18 yrs~~ | ~~Would need to drop CEA~~ | ~~Fear of Childbirth (WDEQ)~~ | ~~yes~~ | ~~Around 18 wks gest.~~ | ~~yes~~ | ~~aOR~~ |  |
| **Intimate Partner Violence** | | | | | | | | | | |
| ~~Barnett (2018)~~ |  |  | ~~CPA, CSA & CEA (CTQ) < 18 yrs~~ |  | ~~IPV (IPV Questionnaire)~~  ~~Past yr exposure to all types of IPV~~ |  | ~~Preg & PP~~ |  | ~~LR~~ | ~~Outcomes not comparable re severities and timing of assessment. Castro is pregnancy whereas Barnett is pregnancy and PP~~ |
| ~~Barrios (2015)~~ | ~~PrOMIS, M = 28.0 (6.2), Peru~~ |  | ~~CPA & CSA (ACES), ≤ 18 yrs~~ |  | ~~Lifetime IPV - qs from health survey~~ |  | ~~9-10 weeks gest.~~ |  | ~~LR~~ | ~~Timing differs and analyses~~ |
| ~~Castro (2003)~~ | ~~Comm, M = 25.0 (5.5), Mexico~~ | ~~Yes for age, no for culture~~ | ~~CPA & CEA part of a larger interview~~ | ~~Yes for CPA only~~ | ~~Pregnancy IPV (Index of Spouse Abuse), lifetime~~ | ~~yes~~ | ~~Preg (not detailed when)~~ | ~~Unsure~~ | ~~PR~~ |  |
| ~~Diestel (2022)~~ | ~~Hispanic, low income highly trauma exposed~~ | ~~No~~ | ~~CTQ (CPA, CSA, CEA)~~ | ~~Yes~~ | ~~IPV (CTS) (past-year)~~ | ~~no~~ | ~~3~~^~~rd~~^ ~~trimester~~ | ~~No~~ |  | ~~Sample not comparable~~ |
| **Mode of delivery** | | | | | | | | | | |
| Lukasse (2010b) | MoBA, Norway 25-35 (79%) |  | CA (Norvold) |  | CS |  | 30-34 weeks |  | LR | Different outcome |
| ~~Lukasse (2011)~~ | ~~MoBA, 25-34 (83%)~~ | ~~yes~~ | ~~CA (Norvold)~~ | ~~Yes~~ | ~~Wish for a CS~~ | ~~No~~ | ~~18-30 weeks~~ | ~~yes~~ | ~~LR~~ | ~~Different outcome~~ |
| ~~Nerum (2013)~~ |  |  | ~~CSA (part of interview)~~ | ~~No~~ | ~~Birth outcomes (medical data)~~ |  |  |  | ~~chi-S~~ | ~~Different predictor~~ |
| Schei (2014) | BIDENS, 30.1 | Yes age | CA (based on Norvold) ≤ 18 yrs | yes | Operative delivery (spontaneous vaginal, elective CS, forceps, emergency CS | Yes for CS | ns | unsure | LR | Not enough studies to compare |
| **Postnatal anxiety** | | | | | | | | | | |
| Malta (2012) | AoB, Canada, 72.6% aged 25-34 |  | Any CA (interview questions) | yes | SSAI |  | <25 weeks gest. |  | LR | Not enough studies to compare |
| Nagl (2017) | Comm, 30.6 (4.5), Germany | Yes for age | CPA, CSA & CEA (CTQ) | yes | SCL-90 (German version) | yes | < 16wks PP, (M = 8.1 (3.2) |  | LR | Not enough studies to compare |
| ~~Mayhew (2022)~~ | ~~COPE, Comm, 33.9(4.3)~~ | ~~Yes for age~~ | ~~CEA (ANRQ)~~ | ~~No~~ | ~~EPD-3A~~ | ~~yes~~ | ~~ns~~ |  | ~~HLR~~ | ~~Predictor different unless we use CEA only for Nagl~~ |
| **Postpartum depression** | | | | | | | | | | |
| ~~Belete (2020)~~ | ~~Comm, 62.3% aged 25-34, Ethiopia,~~ |  | ~~CSA (adapted from interview qs)~~ | ~~No~~ | ~~PPD (EPDS)~~ | ~~Yes~~ | ~~6-8 wks postpartum~~ |  | ~~LR~~ | ~~No studies CSA only and to maintain independence cannot use CSA from others plus assess CA.~~ |
| Bahadur (2021) | M = 26.94 (5.6), Turkey | Yes for age | CPA, CSA (contact only) & CEA (ACES) | Yes for analyses using CA only | PPD (EPDS) | Yes | 1 wk postpartum | With Plaza (timing imp for postnatal dep as levels can change depending on time of assessment | LR | Not enough studies to compare |
| ~~Choi (2018)~~ | ~~28.0 (5.9), comm, UK~~ | ~~Yes for age~~ | ~~CPA, CSA (contact) & CEA (CTQ), ≤18 yrs~~ | ~~Yes for analyses using CA only~~ | ~~PPD (EPDS)~~ | ~~Yes~~ | ~~I yr PP~~ | ~~No~~ | ~~LinR~~ | ~~Timing differs~~ |
| ~~Kiewa (2022)~~ | ~~Aust, AGDS (clinical sample), 39.0 (Mdn)~~ | ~~No~~ | ~~CEA (sgl question)~~ | ~~No~~ | ~~PND (diagnosis or EPDS)~~ | ~~Yes~~ | ~~Various (L)~~ | ~~No~~ | ~~LR~~ | ~~specific sample – depression history. No other studies examine CEA only (as above)~~ |
| ~~Mahenge (2018)~~ | ~~Comm, 67.4% aged 22-35, Tanzania (M = 27.0)~~ | ~~Yes for age~~ | ~~CPA, CSA (contact only), CPY (ACES)~~ | ~~Yes for analyses using CA only~~ | ~~PPD symptoms (PHQ-9)~~ | ~~Yes~~ | ~~1-9mths PP~~ | ~~No~~ | ~~LR~~ | ~~Timing differs~~ |
| Malta (2012) | Canada, 72.6% aged 25-34 | Yes for age | purpose-created, CA ≤ 18 yrs | Yes for analyses using CA only, | Dep (EPDS ≥ 10) | Yes | 4 mths PP | Comparable with Nagl | LR | Not enough studies to compare |
| Nagl (2017) | M = 30.6 (4.5) | Yes for age | CPA, CSA (contact), CEA (CTQ, ≤18 years) | Yes for analyses using CA only | PP dep (BDI) | yes | 16 wks postpartum | With Malta | LR | Not enough studies to compare |
| Plaza (2012) | M = 32.6 (4.6), Spain, comm, | Yes for age | CA (CPA, CSA (contact), CEA [ETI-SR]), ≤ 18 yrs | Yes for analyses using CA only | PPD (EPDS) | Yes | 24-48hrs postpartum | Comparable with Bahadur | LR | Not enough studies to compare |
| ~~Tebeka (2021)~~ | ~~France, 88.6% aged 25-40, IGEDEPP~~ | ~~Yes for age~~ | ~~CPA & CSA (Contact) (CTQ)~~ | ~~No as does not include CEA~~ | ~~PPD (semi-structured interview)~~ | ~~Yes~~ | ~~Just after delivery~~ | ~~yes~~ |  | ~~Abuse differs~~ |
| **Perinatal depression** | | | | | | | | | | |
| ~~Akinbode (2019)~~ | ~~Comm, 25.9 (5.5) US~~ |  | ~~CPA & CSA (contact) (structured interview) < 13 yrs~~ | ~~Yes with Giallo~~ | ~~Dep (EPDS),~~ | ~~yes~~ | ~~6 & 12 wks PP~~ | ~~Yes with Kang & Li, Obgo, R-Blackmore, Khnalari~~ | ~~LinR~~ | ~~Timing differs with Giallo and only two studies to compare~~ |
| ~~Giallo (2017)~~ | ~~MHS, Aust 25-34~~ | ~~Yes for age~~ | ~~CPA & CSA (contact) (CMHSR)~~ | ~~Yes with Akinbode~~ | ~~Dep (EPDS)~~ | ~~Yes~~ | ~~3,6,12mths,PP~~ | ~~R-Blackmore~~ | ~~LR~~ | ~~Timing differs with Akinbode – as above~~ |
| Kang (2022) | Turkey, 95.7% aged 20-39 | Yes for age | CA (sgl question) | Yes with Khanlari & Ogbo & Li | Peripartum dep (EPDS) | yes uses cutoff > or = 10 | Within 4 wks PP | Yes with & Li, Ogbo, R-Blackmore, Khnalari | aOR (compare with Khanlari) | Yes with Li, & Ogbo  Can compare unadjusted model  only for CA measure. Only 2 studies to compare |
| Khanlari (2019) | Aust 20-34, comm | Yes for age | History of childhood abuse (Yes/No) | Yes with Kang & Ogbo & Li | Distress (EPDS, 10–12)  High dep symptoms (≥13). | yes | Within 6 wks PP | Li, Ogbo, R-Blackmore & Kang | aOR (compare with Kang) | Only 2 studies to compare |
| Li (2017) | China comm, 29.0 (4.0) | Yes for age | CPA, CSA (contact), CEA (CTQ) | Yes with Kang & Khanlari & Ogbo | Antepartum dep (EPDS) | yes | 1 & 4 wks PP | Yes with Kang, Ogbo, R-Blackmore, Khanlari | LR (compare with Ogbo) | Only 2 studies to compare |
| Ogbo (2019) | Aust (CALD women), 76% aged 20-34 | Yes for age but no for sample (not comm) | CA (yes/no) | Yes with Kang & Khanlari, Ogbo & Li | Distress (10-12) & dep symptoms <13(EPDS) | yes | 1^st^ prenatal care vists (around 4-6 wks) | Kang, Li, R-Blackmore, Khanlari | LR (compare with Li) | As above |
| ~~Robertson-Blackmore (2013)~~ | ~~Comm (low -inc) 24.5 (3.7)~~ | ~~Yes for age, no for sample~~ | ~~CPA/neglect, CSA (contact) (qs from SCID)~~ | ~~CSA with Akinbode, Giallo, Li~~ | ~~Antepartum dep (SCID)~~ | ~~yes~~ | ~~6-8 wks & 6 mths PP~~ | ~~6-8 wks with Kang, Li, Ogbo, Khanlari;~~ | ~~LR~~ | ~~Sample not comm~~ |
| **Perinatal PTSD** | | | | | | | | | | |
| Lev-Wiesel (2009) | Israel, 30.35 (5.0) | Yes with Sumner | CSA (CSES), < 14 years (contact and non contact) | Yes for CSA only | Birth-related PTSD symptoms (past 2 weeks) (PSS), disassociation (D-HES) | Yes for Sumner | mid-pregnancy and at 2 and 6 months following childbirth |  | chi-S, LinR | Only 2 studies to compare |
| ~~Robertson-Blackmore (2013)~~ | ~~Comm, predominantly low income, inner-city population US, 24.5 (3.7)~~ | ~~No for sample~~ | ~~CPA/neglect, CSA (questions from SCID)~~ | ~~Yes for CSA only~~ | ~~Lifetime diagnosis of PTSD (SCID)~~ | ~~NO~~ |  |  | ~~LR~~ | ~~No for sample~~ |
| Sumner (2012) | Comm (Latina), 27.7 (5.8) | Yes with Lev-Wiesel | CPA, CSA, CEA (ACES & THQ) ≤ 18 yrs | Yes for CSA only | PTSD symptoms (last mth) (PCL-C) | Yes for Lev-Wiesel | during preg, 7 & 13 months PP |  | LinR | Only 2 studies to compare |
| **Preterm birth** | | | | | | | | | | |
| Cammack (2017) | 21.7 | Yes for age | CPA, CSA (contact) & CEA (CTQ) | Overall with M-Zilko | PTB (< 37) very PTB (< 34 wks) | Yes | *n/a* | Yes | LR | Only 2 studies to compare |
| ~~Hyle (1995)~~ | ~~23.4 (4.8), US, Low-income~~ | ~~Yes age no on demographic~~ | ~~CSA (Russel's survey), contact & non-contact ≤ 18 yrs~~ | ~~Broader definition so not comparable~~ | ~~Gestational age (medical data)~~ | ~~Yes~~ | *~~n/a~~* | ~~Yes~~ | ~~LinR~~ | ~~No for sample~~ |
| ~~Leeners (2013)~~ | ~~38.7, Germany, clinical for abuse exposure & control (comm)comm, US~~ | ~~No for age~~ | ~~CSA (unsure?) & CPA (modified Wyatt)~~ | ~~For CSA yes for those denoted *~~  ~~For CPA yes for those denoted #~~ | ~~PTB~~ | ~~yes~~ | *~~n/a~~* | ~~yes~~ | ~~chi-S~~ | ~~No for age~~ |
| Margerison-Zilko (2017) | 90.7% aged 20-34 | Yes for age | CA, 3 qs on CPA/CSA (contact) & witness violence | Overall with Cammack | Early PTB ≤ 34, PTB < 37, late PTB 35-36 wks (medical data) | yes | *n/a* | yes | LR | Only 2 studies to compare |
| **Unintended pregnancy** | | | | | | | | | | |
| ~~Dietz (1999)~~ | ~~prenatal, Hispanic, low income highly trauma exposed~~ | ~~No~~ | ~~CPA, CSA, CPY (CTS & Wyatt)~~ |  | ~~Unintended pregnancy (sgl question)~~ | ~~Yes~~ |  |  |  | ~~No for sample~~ |
| Drevin (2019) |  |  | CPA, CSA & CEA (ACES) |  | Unplanned pregnancy | Yes |  |  | aRR | Only 2 studies to compare |
| Lukasse (2015) | Norway |  | CA, CPA, CSA, CEA (Norvold), ≤ 18 years |  | Unintended pregnancy | yes |  |  | aOR | Only 2 studies to compare |

*Note*. ACES = Adverse Childhood Experiences Scale, aOR = adjusted odds ratio, aRR = adjusted risk ratio, BDI = Beck Depression Inventory, CA = child abuse, CAR = Cortisol Awakening Response, CEA = child emotional abuse, CESD = Center for Epidemiologic Studies Depression Scale, Chi-S = chi-square, CMHSR = Child Maltreatment History Self Report, comm = community, CPA = child physical abuse, CPS = child protection services, CPSAQ = Childhood Physical and Sexual Abuse Questionnaire, CPY = child psychological abuse, CS = cesarian, CSA = child sexual abuse, CSES = Childhood Sexual Experiences Scale, CTQ = Child Trauma Questionnaire, CTS = Conflict Tactics Scales, Dep = depression, EPDS = Edinburgh Postnatal Depression Scale, ETI = Early Trauma Inventory, gest = gestation, HLR = Hierarchical linear regression, IPV = intimate partner violence, LinR = Linear regression, LR = logistic regression, MoBA = Norwegian Mothers and Babies Cohort study, Norvold = Norvold Abuse Questionnaire, PCL-C = Posttraumatic Stress Disorder Checklist Civilian Version, PHQ-9 – Patient Health Questionnaire-9, PICS = positive influences in childhood, PP = postpartum, PPD = postpartum depression, PR = prevalence ratio, preg = pregnancy, PSS = posttraumatic stress disorder symptom scale, PSQ = Personal Safety Questionnaire, PTB = preterm birth, PTSD = Post Traumatic Stress Disorder, q = question, S-R = self report, SCID = Structured Clinical Interview for DSM-5, SES = socioeconomic status, sgl = single, THQ = Trauma History Questionnaire, US = United States of America, qs = questions, WEDQ = Wijma Delivery Expectancy Questionnaire
