# Supplementary material for: Childhood abuse and perinatal outcomes for mother and child: A systematic review of the literature
Source: PLoS One. 2024 May 24;19(5):e0302354. doi: 10.1371/journal.pone.0302354 (PMC11125509; doi:10.1371/journal.pone.0302354)
Supplement: S2 Table — (DOCX) [file pone.0302354.s003.docx]

| **Supplementary Table 2**  *Overview of the included studies* | | | |
| --- | --- | --- | --- |
| **Developed countries** | | **Child abuse measures** |  |
| USA | 36 | Purpose created/adapted/single questions/composite | 28 |
| Norway | 10 | Childhood Trauma Questionnaire | 23 |
| Australia | 8 | Adverse Childhood Experiences Survey | 16 |
| Germany | 6 | Norvold Abuse Questionnaire | 6 |
| Canada | 4 | Structured Interview/part of larger interview | 6 |
| Israel | 2 | Conflict Tactics Scale | 4 |
| Iceland* | 2 | Childhood Sexual Experiences Scale | 1 |
| Spain | 2 | Early Trauma Inventory | 2 |
| Turkey | 1 | Adult Attachment Interview | 1 |
| UK | 2 | Antenatal Risk Questionnaire | 1 |
| Belgium* | 1 | Childhood Experience of Care & Abuse Questionnaire | 1 |
| Denmark* | 1 | Childhood Experiences of Violence Questionnaire | 1 |
| Estonia* | 1 | Childhood Physical and Sexual Abuse Questionnaire | 1 |
| France | 1 | Childhood Sexual Assaults Scale | 1 |
| Korea | 1 | Life Stressors Checklist | 1 |
| Sweden* | 1 | Personal Safety Questionnaire | 1 |
| The Netherlands | 1 | Trauma History Questionnaire | 1 |
| **Developing countries** | | **Risk of bias** |  |
| Peru | 6 | Low | 3 |
| South Africa | 4 | Low-Medium | 65 |
| China | 3 | Medium | 13 |
| Brazil | 2 | Medium-High | 14 |
| Mexico | 2 |  |  |
| Ethiopia | 1 |  |  |
| Kenya | 1 |  |  |
| Tanzania | 1 |  |  |

*Note.* * = BIDENS study. Studies that used the same samples/ measures were excluded.
