# Supplementary material for: Childhood abuse and perinatal outcomes for mother and child: A systematic review of the literature
Source: PLoS One. 2024 May 24;19(5):e0302354. doi: 10.1371/journal.pone.0302354 (PMC11125509; doi:10.1371/journal.pone.0302354)
Supplement: S3 Table — (DOCX) [file pone.0302354.s004.docx]

| **Table 3**  *Characteristics of included studies* | | | | | | | | | | |
| --- | --- | --- | --- | --- | --- | --- | --- | --- | --- | --- |
| **Study (first author, year)** | **Country** | **Design** | **Sample** | **Participants** | **Mean age (SD)** | **Abuse type (exposure %)** | **Age** | **CA Measure** | **Outcome measures** | **QR** |
| Akinbode (2019) | US | L | comm | 197 perinatal | 25.9 (5.5) | CPA & CSA | 13 | Structured Interview | Dep (EPDS)  Anxiety (STAI) | 10/M |
| Ansara (2005) | US | C-S | comm | 200 postnatal | 31.7 (5.0) | CPA, CSA, CEA | 14 | CTS (I) | General health (qs on 13 health symptoms) | 8/M |
| Appleyard (2011) | US | L | comm | 199 prenatal | 27.3 (5.9) | CPA, CSA | *n/s* | adapted from CTS (I) | Alcohol use (custom); offspring victimization (CPS records) | 9.5/M |
| Atkinson (2021) | US | C-S | Add Health | 1059 postnatal | 18-26 years | CPA | 6^th^ grade | Purpose created (I) | Stillbirth, miscarriage,  abortion (single q) | 8/M |
| Bahadur (2021) | Turkey | C-S | comm | 900 postnatal | 26.9 (5.6) | CPA (1.3), CSA0.1), CEA2.1) | *n/s* | ACES (I) | Dep (EPDS), Social Support (MSPSS) | 9/L-M |
| Barnett (2018) | SA | L | DCHS | 832 prenatal | 26.2 | CPA (23), CSA (16), CEA (32) | 18 | CTQ | IPV (Intimate Partner Violence Questionnaire) | 8/M |
| Barrios (2015) | Peru | C-S | PrOMIS | 1521 prenatal | 28.0 (6.2) | CPA (37), CSA (8), CPA/CSA (24) | 18 | ACES (S-R) | Dep (PHQ-9); Lifetime IPV; General health (adapted version) | 10.5/L |
| Belete (2020) | Eth | C-S | comm | 988 postnatal | *n/s* | CSA (13) | 18 | Adapted qs (I) | Postpartum dep (EPDS) | 8/M |
| Benedict (1999) | US | C-S | comm | 357 prenatal, nulliparous | 20-24 years | CSA (37) | 19 | Adapted S-R qs | Dep (CESD); PTB birth & birthweight (medical data) | 9/M |
| Bert (2009) | US | C-S | PFTP | 681 postnatal, nulliparous | 19.8 | CPA, CSA & CEA | *n/s* | CTQ (I) | CA potential; parenting knowledge &style (KIDI), philosophy (CAPI) | 9/L-M |
| Blalock (2011) | US | C-S | Smoking study | 201 perinatal | 25.0 | CPA (21), CSA (33), CEA (32) | 12 | CTQ (I) | Smoking (WISDM-68) | 8/M |
| Brunst (2017) | US | L | ACCESS | 857 perinatal | 27.0 | CPA/CSA (25) | 17 | CTS (S-R) | Offspring asthma (diagnosis) | 11/L-M |
| Brunton (2020) | Aust | C-S | comm | 638 prenatal | 26.5 (5.2) | CPA (68), CSA (28), CPY (42) | 18 | ACES & CEVQ (S-R) | Pregnancy-related anxiety (PrAS) | 11/L-M |
| Bublitz (2014) | US | C-S | BAMBI | 185 prenatal | 26.5 (5.5) | CPA & CSA | 18 | ACES (S-R) | CAR (saliva), Family functioning (Family Assessment Device) | 6/M-H |
| Bublitz (2012) | US | C-S | BAMBI | 135 prenatal | 26.0 (6.0) | CPA & CSA | 18 | ACES (S-R) | CAR (salivary) | 6/M-H |
| Bublitz (2022) | US | L | Sleep apnea study | 98 prenatal | 30.0 (5.0) | CPA (16), CSA (20) & CEA (25) | 18 | ACES (S-R) | Neutrophil-lymphocyte ratio (medical chart) | 10.5/L-M |
| Cammack (2017) | US | L | EWMHP NLSAAH | 4181 perinatal | 21.7 | CPA (44), CSA (23), CEA (22) | 18 | CTQ (S-R) | Smoking (S-R); PTB (< 37 weeks) very PTB (< 34 weeks); Dep (CESD) | 10/L-M |
| Cammack (2011) | US | L | comm | 312 prenatal | *ns* | CPA (17), CSA (19), CEA (16) | *n/s* | CTQ (S-R) | Persistent bacterial vaginosis | 8/M-H |
| Castro (2003) | Mex | C-S | comm | 914 prenatal | 25.0 (5.5) | CPA & CEA | *n/s* | Part of larger interview | Pregnancy IPV (Index of Spouse Abuse) | 6/M-H |
| Choi (2018) | UK & SA | L | ERLTS & comm | 1166 prenatal | 27.2* | CPA (7.5*), CSA (8.5*) & CEA (10*) | 18 | CTQ (I) | Mother-infant bonding, (I); Dep (EPDS); Child exposure to harm (I) | 8.5/M |
| Chung (2008) | US | C-S | comm | 1984 prenatal | 24.0 (6.0) | CPA (52), CSA (13), CEA (27) | 18 | ACES (I) | Dep (CESD) | 11/L |
| Cohen (2002) | SA | L | comm | 200 postnatal | 18-40 | CSA&CEA (14) | 14 | Adapted qs (I) | Dep (EPDS) | 8.5/M |
| Coles (2016) | Aust | C-S | ALWHS | 3778 women (retrospective) | 28.5 (3.5) | CSA (16) | *n/s* | single q (S-R) | Breastfeed = single question | 9/M |
| Corona (2022) | US | C-S | MADRES | 382 prenatal, Hispanic, most low inc. | 28.6 (6.0) | CA (63) | *n/s* | ACES (I) | Dep (CES-D) | 9.5/L-M |
| Cowell (2020) | US | C-S | PRISM | 628 prenatal | 28.7 (9.2) | CPA (38), CSA (9), CEA (63) | 11 | CTQ (S-R) | PTB | 10/L-M |
| Diestel (2022) | US | C-S | IPV & epigenetic risk study | 198 prenatal, Hispanic, low inc. highly trauma exposed | 26.6 (5.3) | CPA, CSA &, CEA | *n/s* | CTQ | PTSD symptoms (STRESS-A), IPV (CTS) | 9/L-M |
| Dietz (1999) | US | C-S | ACEs study | 1193 women (retrospective) | 20-50 | CPA (52), CSA (29), CPY (64) | 18 | CTS & Wyatt’s measure | Unintended preg (single q) | 9.5/L-M |
| Drevin (2019) | NY | L | MCCS | 76197 prenatal | 30.7 | CPA (5), CSA (7), CEA (14) | *n/s* | ACES (S-R) | Unplanned preg (single q) | 10.5/L-M |
| Eide (2010) | NY | C-S | MCCS | 58139 prenatal | *n/s* | CPA (2), CSA (3), CPA/CSA (2) | 18 | Norvold (S-R) | Baby’s health worries (single q) | 10/L-M |
| Elfgen (2017) | GY | C-S | comm | 255 pre-and postnatal | 38.7 | CSA | 18 | Modified scale | Breastfeed (10 qs) | 5.5/ M-H |
| Farre-Sender (2018) | Spain | L | comm | 251 pre-and postnatal | 34.0 (4.8) | CPA, CSA & CEA | *n/s* | ETI (S-R) | Mother-infant bonding (Postpartum Bonding Questionnaire) | 10/M |
| Finy & Christian (2018) | US | C-S | comm | 214 prenatal | 29.4 (4.9) | CPA, CSA & CEA | *n/s* | CTQ | C-reactive protein & interleukin-6 | 9/M |
| Freedman (2017) | US | C-S | SCRN & OASIS | 633 prenatal | 89.8% aged 20-39 | CPA (40), CSA (47), CEA (64) | *n/s* | CTQ (I) | Stillbirth | 12/L |
| Galbally (2019) | Aust | L | MPEWS | 246 postnatal | 31.4 (4.6) | CPA (43), CSA (41), CEA (69) | *n/s* | CTQ | Dep diagnosis (SCID); Perinatal dep symp (EPDS); Parenting stress (PSI) | 8/M |
| Gelaye (2016) | Peru | C-S | PrOMIS | 2970 prenatal | 28.1 (6.3) | CPA (39), CSA (16) | 18 | ACES (I) | Migraine (ICD Headache Disorders). | 11/L-M |
| Gelaye (2015) | Peru | C-S | PrOMIS | 630 prenatal | 28.8 (6.6) | CPA (41), CSA (8), CPA/CSA (26) | *n/s* | ACES (I) | Stress-related sleep disturbance (Ford Insomnia Response to Stress); Preg sleep quality (PSQ); Dep (PHQ-9) | 11.5/L |
| Giallo (2017) | Aust | L | MHS | 1507 perinatal, nulliparous | 25-34 | CPA & CSA | *n/s* | Ever exp CPA or CSA (S-R) | Dep symp (EPDS) | 8.5/L |
| Heimstad (2006) | NY | C-S | comm | 1452 prenatal | 29.6 | CPA (10), CSA (8) | *n/s* | Sgl q (S-R) | FoC (WDEQ); Complicated delivery | 7.5/M |
| Huth-Bocks (2013) | US | C-S | comm | 120 prenatal | 26.0 (5.7) | CPA, CSA & CEA | *n/s* | CTQ (I) | IPV (CTS); PTSD symptoms (PCL-C) | 9/M |
| Hyle (1995) | US | C-S | low-inc. | 241 postnatal | 23.4 (4.8) | CSA | 18 | Russel's survey (I) | Birthweight; gestational age | 9/M |
| Jantzen (1998) | US | C-S | comm | 1189 prenatal | 24.3 | CPA (13), CSA (10) | 17 | Purpose created (I) | Lifetime cocaine use (S-R & urine) | 8/M |
| Kang (2022) | Seoul, Korea | C-S | SHFSP | 80116 | 95.7% aged 20-39 | CA (3.4) | *n/s* | Sgl q | Dep (EPDS), psychosocial health (purpose created) | 9.5/L-M |
| Khanlari (2019) | Aust | L | comm | 53032 postnatal (retrospective) | 20-34 | CA (13*) | *n/s* | Sgl q (I) | Distress (EPDS, 10–12); High dep symptoms (EPDS ≥13). | 9.5/L-M |
| Kiewa (2022) | Aust | C-S | AGDS | 15198 postnatal, MDD diagnosis | 39.0 (Mdn) | CEA | *n/s* | Sgl q (S-R) | Dep (EPDS) | 11/L-M |
| Kunseler (2016) | Netherlands | L | Generations study | 243 perinatal, nulliparous | 28.2 (5.8) | CPA (19), CSA (2), CPA/CSA (1) | *n/s* | AAI (I) | Parenting self-efficacy (Pictographic Visual Analogue Scale) | 9/M |
| Leeners (2016) | GY | C-S | Cases - clinical | 255 prenatal | 38.7 | CSA (33) | 18 | Modified scale (I) | Obstetric outcomes (medical data) | 7/M |
| Leeners (2010) | GY | C-S | as above | 255 prenatal | 38.7 | CSA | 18 | as above | Preg complications, e.g. hypertension, diabetes | 7/M |
| Leeners (2013) | GY | C-S | as above | 255 prenatal | 38.7 | CSA | 18 | as above | Obstetric & neonatal outcomes | 6/M-H |
| Lehnig (2019) | GY | C-S | comm | 725 postnatal | 30.6 (4.5) | CPA (8), CSA (11), CEA (18) | *n/s* | CTQ (S-R) | Mother-infant bonding (abridged postpartum bonding questionnaire) | 10.5/L-M |
| Lev-Wiesel (2009) | Israel | L | comm | 837 perinatal | 30.4 (5.0) | CSA | 14 | CSES (I) | PTSD symptoms (PSS); Disassociation (D-HES) | 10/L-M |
| Li (2017) | China | L | comm | 260 perinatal | 29.0 (4.0) | CPA (6), CSA (6), CEA (6) | *n/s* | CTQ | Dep (EPDS) | 10.5/L-M |
| Littleton (2015) | US | C-S | comm | 407 prenatal | 27.0 (5.9) | CSA (9) | 14 | 2 adapted qs | Dep (CESD); somatic complaints (PHQ-15) | 9/M |
| Lukasse (2015) | NY | C-S | BIDENs | 7102 prenatal | 73.0% aged 25-31 | CPA, CSA, CEA | 18 | Norvold (S-R) | Unintended preg (single q) | 8.5/L-M |
| Lukasse (2009) | NY | C-S | MCCS | 55776 prenatal | *n/s* | CPA (6), CSA (7), CEA (14) | 18 | Norvold (S-R) | Common complaints in preg (asked if experienced common complaints) | 8.5/L-M |
| Lukasse (2010a) | NY | C-S | BIDENs | 2365 prenatal | 78.7% aged 25-35 | CPA (11), CSA (12), CEA (11) | 18 | Norvold (S-R) | FoC (WDEQ); Dep (EPDS) | 10/L-M |
| Lukasse (2010b) | NY | L | MoBA | 26923 postnatal, primiparous | 83.6% aged 25-34 | CA (19), CPA (5), CSA(6), CPA&CSA(3) | 18 | Norvold (S-R) | Caesarian section | 8.5/L |
| Lukasse (2011) | NY | L | MoBA | 4876 prenatal | *n/s* | CA (21), CPA (6), CSA (7), CEA (16) | *n/s* | Norvold (S-R) | Fear of Childbirth; Caesarian section (MoBA questionnaire) | 8.5/L |
| Lydsdottir (2019) | Iceland | L | comm | 521 perinatal | 28.9 (5.3) | CPA (13), CSA (22) | 17 | ACES (I) | CMD (anxiety [DASS]; Dep [EPDS]) | 6.5/M-H |
| Madigan (2017) | Canada | L | KFPS | 501 postnatal | 32.7 (4.9) | CPA (20), CSA (10) | *n/s* | CEVQ (I) | Biomedical & psychosocial risk (various questionnaires) | 11/L-M |
| Mahenge (2018) | Tanzania | C-S | comm | 500 postnatal | 27.0 | CPA (33), CSA (12), CPY (26), CPA/CSA (39) | *n/s* | ACES (I) | PP Dep symptoms (PHQ-9); IPV (WHO IPV Scale) | 10/L-M |
| Malta (2012) | Canada | L | AoB | 1319 postnatal | 72.6% aged 25-34 | Any CA | 18 | Custom measure (I) | Anxiety (STAI); Stress (PSS); Dep (EPDS ≥ 10); Parenting moral (Parenting Moral Index) | 7.5/M |
| Margerison-Zilko (2017) | US | C-S | POCHS | 2559 prenatal | 90.7% aged 20-34 | CA (CPA/ CSA/ witness violence ([17]) | *n/s* | 3 qs. (S-R) | PTB: early (≤ 34 weeks); preterm (< 37 weeks); late (35-36 weeks) | 9.5/L-M |
| Mason (2016) | US | C-S | NHS | 45500 prenatal, retrospective | *n/s* | CPA (42), CSA (44) | 17 | CTS | Gestational diabetes (diagnosis) | 10/L-M |
| Mayhew (2022) | Aust | L | COPE archival | 240 perinatal | 33.9 (4.3) | CEA (8) | *n/s* | ANRQ | Postnatal anxiety (EPDS, anxiety subscale) | 7.5/M |
| McNaughton Reyes (2020) | SA | L | HIV positive | 1154 perinatal | 25.5 | CA (CSA&CPA [5]) | 12 | 2 qs | Emotional distress (Hopkins Checklist); IPV (Violence against Women Instrument) | 7.5/M |
| Mitro (2019) | Peru | C-S | PAGES | 1327 postnatal | 28.4* | CPA (35), CSA (2), CPA/CSA (5) | 18 | purpose created | Placental abruption | 8/L-M |
| Nagl (2017) | GY | C-S | comm | 741 postnatal | 30.6 (4.5) | CPA (8), CSA (12) CEA (18) | 18 | CTQ | Postpartum dep (BDI); Anxiety (Symptom Checklist 90-Revised) | 11/L |
| Nerum (2013) | NY | C-S | Clinical | 373 prenatal | *n/s* | CSA (27) | 16 | Part of larger interview | Birth duration and outcome (medical records) | 10/M |
| Nieto (2017) | Mex | L | Clinical | 156 postnatal | 27.1 (5.9) | CSA | 17 | CECAQ (I) | Maternal attachment (Maternal Postnatal Attachment Questionnaire) | 6.5/M-H |
| Ogbo (2019) | Aust | L | CALD | 25407 prenatal | 76.1% aged 20-34 | CA (15) | *n/s* | (I) | Distress (EPDS, 10–12); Dep symptoms (EPDS ≥ 13) | 9.5/M |
| Oliveira (2017) | Brazil | C-S | HRP | 456 postnatal | 60.7% aged 20-35 | CSA (9) | *n/s* | THQ (I) | PTSD symptoms (PCL-C) | 9/M |
| Plaza (2012) | Spain | C-S | Comm | 236 postnatal | 32.6 (4.6) | CPA(13), CSA(16), CEA (14), CPA&CEA (6) | 18 | ETI (I) | PPD (EPDS); Free Thyroxin and thyroid-stimulating hormone | 8/M |
| Racine (2018) | Canada | L | AoF | 1994 prenatal | 30.9 (4.4) | CPA (42), CSA (13) | 18 | ACES (S-R) | Reproductive health history; Complications & psychosocial difficulties (custom measure) | 9/M |
| Racine (2020) | Canada | C-S | AoF | 1994 prenatal | 30.9 (4.4) | CPA (42), CSA (13) | 18 | ACES (S-R) | S-R alcohol, drugs, smoking | 8/M |
| Ranchod (2016) | US | C-S | NLSY | 6199 prenatal | 14-22 | CPA (7) | 18 | Single q (S-R) | Preg weight gain (pre-preg less delivery weight) | 10/M |
| Rich-Edwards (2011) | US | C-S | ACCESS & Viva | 3637 prenatal | Viva, 86.1%, 25-40 yrs, ACCESS 75.3%, 20-35 yrs | CPA & CSA (43) | 17 | PSQ | Dep (EPDS) | 9.5/M |
| Roberts (2013) | US | L | NHS | 52498 perinatal | *n/s* | CPA & CEA (65), CSA (34) | 17 | CTQ, 5 qs (S-R) | Child autism (diagnosis) | 9/L-M |
| Robertson-Blackmore (2013) | US | L | comm (low-inc.) | 374 prenatal | 24.5 (3.7) | CPA/neglect (4), CSA (12) | *n/s* | Qs from SCID | Lifetime PTSD; Dep (SCID) | 10/L-M |
| Samia (2020) | Kenya | L | comm | 215 prenatal | 30.6 (4.3) | CPA (78), CSA (18), CEA (51) | *n/s* | ACE-IQ | PrA (10-item scale); Dep (EPDS); Sress (Perceived Stress Scale) | 9/M |
| Sanchez (2017) | Peru | C-S | PrOMIS | 2928 prenatal | 18-49 | CPA (39), CSA (8), CPA & CSA (25) | *n/s* | ACES (S-R) | PTSD symptoms (PCL-C) | 9/M |
| Schei (2014) | various | C-S | BIDENS | 3308 prenatal | 30.1 | CA (23) | 18 | based on Norvold (S-R) | Mode of delivery (Cesarean, operative, emergency) | 8/M |
| Schreier (2015) | US | C-S | PRISM | 180 prenatal | 26.9 (8.1) | CPA, CSA, CEA | 11 | CTQ | Cortisol (hair samples) | 7/M |
| Seng (2014) | US | C-S | STACY | 1581 prenatal, nulliparous | 18-47 | CPA (10), CSA (14) & CEA/neglect (4) | *n/s* | LSC | PTSD symptoms /diagnosis (National Women’s Study PTSD module); Disassociation Experiences Scale | 12/L |
| Senior (2005) | UK | C-S | AVON | 10,641 prenatal | 28.2 | CPA, CSA, CEA | *n/s* | Single q (S-R) | Lifetime and antenatal eating disorder (EDE-Q) | 8/M |
| Shamblaw (2021) | US | L | NESARC | 1279 perinatal | 24.5 (3.7) | CPA (19), CSA (16), CEA (13) | 18 | CTS | Perinatal & obstetric complications (2 qs) | 9.5/L-M |
| Sorbo (2015) | NY | L | MoBa | 51101 perinatal | 77.1% aged 25-34 | CA (CPA, CSA, CEA [18]) | 18 | adapted qs | Breastfeeding cessation (3 qs) | 9/M |
| Stark Stigger (2020) | Brazil | L | Clinical | 647 prenatal | 64.1% < 29 yrs | CPA (3), CSA (3), CEA (4) | *n/s* | CTQ | Maternal-Fetal attachment Scale | 9/L |
| Stephens (2021) | US | L | MMS | 178 prenatal | 30.1 (5.1) | CPA, CSA, CEA | *n/s* | CTQ (S-R) | CAR (saliva), 30 & 60 mins awake | 8.5/L-M |
| Sumner (2012) | US | L | Latina | 194 perinatal | 27.7 (5.8) | CPA, CSA, CEA | 18 | ACES & THQ | PTSD symptoms (PCL-C) | 9/M |
| Swanson (2014) | US | C-S | MACY | 173 postnatal | 28.3 (5.6) | CPA, CSA | *n/s* | CTQ (I) | Maternal sleep (Postpartum Depression Screening Scale) | 9.5/M |
| Tebeka (2021) | France | L | IGEDEPP | 2362 postnatal | 88.6% aged 25-40 | CPA, CSA |  | CTQ | PPD (semi-structured interview) | 12/L |
| Yampolsky (2010) | Israel | C-S | comm | 1830 prenatal | 30.3 (4.9) | CSA (32) | n/s | CSAS (I) | HRP (Dep [CESD], PTS [PSS-I], gynecological problems) S-R | 8.5/M |
| Zhang (2020) | China | C-S | comm | 1825 prenatal | 31.1 (4.4) | CA (8), CPA (5), CSA (5), CEA (3) | *n/s* | CTQ (S-R) | Suicide ideation (PHQ-9); Dep (PHQ-8) | 10.5/L-M |
| Zhang (2021) | China | C-S | As above | As above | 31.1 (4.4) | CPA, CSA, CEA | *n/s* | CTQ (S-R) | Subjective memory impairment (PRMQ) | 10.5/L-M |
| Zhong (2016) | Peru | C-S | comm | 2964 prenatal | 28.1 (6.3) | CA (CPA & CSA [71.8]) | 18 | CPSAQ (I) | Lifetime IPV (adapted measures), Dep (PHQ-9), Suicide ideation (PHQ-9) | 10.5/L-M |

*Note.* AAI = Adult Attachment Interview, ACCESS = Asthma Coalition on Community Environment and Social Stress, ACES = Adverse Childhood Experiences Scale, AGDS = Australian Genetics of Depression Study, ALWHS = Australian Longitudinal Women’s Health Study, ANRQ = Antenatal (Psychosocial) Risk Questionnaire, AoB = All our Babies, AoF = All our Families, Aust = Australia, BAMBI = Behavior and Mood in Mothers, Behavior in Infants, BDI = Beck Depression Inventory, BIDENS = Belgium, Iceland, Denmark, Estonia, Norway, and Sweden cohort study, C-S = cross-sectional, CA = child abuse, CALD = Culturally and Linguistically Diverse, CAPI = Child Abuse Potential Inventory, CAR = Cortisol Awakening Response, CEA = childhood emotional abuse, CECAQ = Childhood Experience of Care & Abuse Questionnaire, CESD = Center for Epidemiologic Studies Depression Scale, CEVQ = Childhood Experiences of Violence Questionnaire, CMD = common mental disorders, comm = community sample, COPE = Centre of Perinatal Excellence, CPA = childhood physical abuse, CPS = child protection services, CPSAQ = Childhood Physical and Sexual Abuse Questionnaire, CPY = childhood psychological abuse, CSA = childhood sexual abuse, CSAS = Childhood Sexual Assaults Scale, CSES = Childhood Sexual Experiences Scale, CTQ = Child Trauma Questionnaire, CTS = Conflict Tactics Scales, DASS = Depression, Anxiety & Stress Scale, DCHS = Drakenstein Child Health Study, Dep = depression, D-HES = Hebrew version of the Dissociative Experiences Scale, EDE-Q = Eating Disorder Examination Questionnaire, EPDS = Edinburgh Postnatal Depression Scale, ERLTS = Environmental Risk Longitudinal Twin Study, Eth = Ethiopia, ETI = Early Trauma Inventory, EWMHP = Emory Women’s Mental Health Program, GY = Germany, HRP = high risk pregnancy, I = interview, IGEDEPP = Interaction of Gene and Environment of Depression during Postpartum, inc. = income, IPV = intimate partner violence, KFPS = Kids, Families, Places Study, KIDI = Knowledge of Infant Development Inventory – Short Form, L = longitudinal, LSC = Life Stressors Checklist, MACY = Maternal Anxiety during the Childbearing Years, MADRES = Maternal and Development Risks from Environmental and Social Stressors pregnancy cohort, MCCS = Mother and Child Cohort Study, MDD = Major Depressive Disorder, Mdn = Medium, Mex = Mexico, MHS = Maternal Health Study, MMS = Measurement of Maternal Stress, MoBA = Norwegian Mothers and Babies Cohort study, MPEWS= Mercy Pregnancy & Emotional Wellbeing Study, MSPSS = Multidimensional Scale of Perceived Social Support, NESARC = National Epidemiologic Survey on Alcohol and Related Conditions, NHS = Nurse’s Health Study, NLSAAH = National Longitudinal Study of Adolescent to Adult Health, NLSY = National Longitudinal Survey of Youth, Norvold = Norvold Abuse Questionnaire, n/s = not stated, NY = Norway, OASIS = SCRN-Outcomes after Study Index Stillbirth, PAGES = Placental Abruption Genetic Epidemiology Study, PCL-C = Posttraumatic Stress Disorder Checklist Civilian Version, PFTP = Parenting for the First Time Project, PHQ-9 – Patient Health Questionnaire-9, POCHS = Pregnancy Outcomes and Community Health study, PPD = Postpartum depression, PrA = Pregnancy-related Anxiety, PrAS = Pregnancy-related Anxiety Scale, preg = pregnancy, PRISM = Programming of Intergenerational Stress Mechanism study, PRMQ = Prospective and Retrospective Memory Questionnaire, PrOMIS = Pregnancy Outcomes, Maternal and Infant Study, PSI = Parenting Stress Index Short Form, PSQ = Personal Safety Questionnaire, PSS = posttraumatic stress disorder symptom scale, PTB = preterm birth, PTS = Post Traumatic Stress, PTSD = Post Traumatic Stress Disorder, q = question, QR = quality rating, S-R = self report, SA = South Africa, SCRN = Stillbirth Collaborative Research Network, SCID = Structured Clinical Interview for DSM-5, SHFSP = Seoul Healthy First Step Project, sgl = single, SRQ-20 = Self-reporting questionnaire, STACY = Stress, Trauma, Anxiety, and the Childbearing Year Project, STAI = State Trait Anxiety Inventory, THQ = Trauma History Questionnaire, UK = United Kingdom, US = United States of America, WEDQ = Wijma Delivery Expectancy Questionnaire, * indicates weighted averaged value reported.
