# Supplementary material for: Childhood abuse and perinatal outcomes for mother and child: A systematic review of the literature
Source: PLoS One. 2024 May 24;19(5):e0302354. doi: 10.1371/journal.pone.0302354 (PMC11125509; doi:10.1371/journal.pone.0302354)
Supplement: S4 Table — (DOCX) [file pone.0302354.s005.docx]

| **Table 4**  *Main findings of included studies* | | | | | | |
| --- | --- | --- | --- | --- | --- | --- |
| **Study (first author, year)** | **Main outcome examined** | **Covariates** | **Analysis** | **Primary Results** | **Ref grp** |  |
| Akinbode (2019) | Dep & anxiety | race, demos | Lin. Reg. | **Dep**  CSA predicts prenatal dep, β = 2.41 [0.60, 4.21]**  CPA predicts prenatal dep, β = 3.36 [0.39, 6.34] *  CSA & CPA predicts prenatal dep, β = 3.12 [0.34, 5.89] *  CSA predicts postnatal dep (6 wks), β = 2.23 [0.33,3.91] *  CSA predicts postnatal dep (12 wks), β = 3.48 [1.93, 5.04] ***  CPA predicts postnatal dep (12 wks), β = 2.91 [0.35, 5.46] *  **Anxiety**  CSA predicts prenatal anxiety, β = 6.08 [2.50, 9.66] ***  CSA predicts postnatal anxiety (6 wks), β = 3.97 [0.46, 7.49] *  CSA predicts postnatal anxiety (12 wks), β = 5.31 [2.02, 8.60] ** | *n/a* | |
| Ansara (2005) | Common health problems | psychosocial & psychological variables | Log. Reg. | CSA inc. the odds of backache, OR= 2.36 [0.98, 5.65]* | NA | |
| Appleyard (2011) | Maternal substance use & offspring victimization | maternal mental health | SEM | CPA predicts offspring victimization, mediated by substance abuse (the mediated effect was assoc. with a .26 SD change in the likelihood of victimization)*  CSA predicts offspring victimization, mediated by substance abuse (the mediated effect was assoc. with a .19 SD change in the likelihood of victimization)* | *n/a* | |
| Atkinson (2021) | Preg outcomes (stillbirth, miscarriage & abortion) | race, chronic disease, substance use | HLR | No significant results | NA | |
| Bahadur (2021) | PPD & social support | age, perceived SES, & educational level | Log. Reg. | CEA inc. the odds of PPD, aOR = 6.70 [2.23, 20.08]***  CPA & CSA were *ns*. | NA | |
| Barnett (2018) | IPV | sociodemographics | Log. Reg. | **Emotional IPV**  CSA inc. the odds of high emotional IPV, aOR = 4.28 [1.48, 12.39)**  CEA inc. the odds of high emotional IPV, aOR = 4.31 [1.67,11.13]**  CPA inc. the odds of high emotional IPV, aOR = 3.16 [1.22, 8.18]*  CPA inc. the odds of moderate emotional IPV, aOR = 2.01 [1.10, 3.69]*  **Phy IPV**  CSA inc. the odds of high phy IPV, aOR = 5.86 [2.63,13.08]***  CEA inc. the odds of high phy IPV, aOR = 2.89 [1.33, 6.30]**  CPA inc. the odds of high phy IPV, aOR = 4.95 [2.10, 11.70]***  **Sexual IPV**  CSA inc. the odds of high/moderate sexual IPV, aOR = 5.44 [2.43, 12.19]***  CEA inc. the odds of high/moderate sexual IPV, aOR = 3.31 [1.60, 6.94]***  CPA inc. the odds of high/moderate sexual IPV, aOR = 4.54 [2.07, 9.94]***  **Combined IPV**  CSA inc. the odds of high combined IPV aOR = 2.97 [1.50, 5.91]**  CEA inc. the odds of high combined IPV, aOR = 2.46 [1.31, 4.61]**  CPA inc. the odds of high combined IPV, aOR = 3.61 [1.85, 7.01]***  CPA inc. the odds of moderate combined IPV, aOR = 2.23 [1.26, 3.93]** | Low /no IPV | |
| Barrios (2015) | IPV, poor health & dep | a = IPV in the current preg  b = demos, parity, financial stress | Log. Reg | **Lifetime IPV (LIPV)**  CA (any CPA or CSA) inc. the odds of LIPV, aOR = 2.20 [1.72, 2.83]^b^*  CA inc. the odds of phy LIPV, aOR = 1.94 [1.45, 2.58]^b^*  CA inc. the odds of sexual LIPV, aOR = 2.27 [1.30, 3.95]^b^*  CA inc. the odds of phy & sexual LIPV, aOR = 3.29 [2.01, 5.38]^b^*  CPA inc. the odds of LIPV, aOR = 1.57 [1.19, 2.07]^b^*  CPA inc. the odds of phy LIPV, aOR = 1.63 [1.19, 2.24]^b^*  CSA inc. the odds of LIPV, aOR = 2.14 [1.39, 3.29]^b^*  CSA inc. the odds of phy LIPV, aOR = 1.88 [1.13, 3.13]^b^*  CSA inc. the odds of phy & sexual LIPV, aOR = 3.36 [1.59, 7.10]^b^*  CPA & CSA inc. the odds of LIPV, aOR = 3.73 [2.75, 5.04]^b^*  CPA & CSA inc. the odds of phy LIPV, aOR = 2.68 [1.88, 3.83]^b^*  CPA & CSA inc. the odds of sexual LIPV, aOR = 5.00 [2.71, 9.22]^b^*  CPA & CSA inc. the odds of phy & sexual LIPV, aOR = 7.14 [4.15, 12.26]^b^*  **Past year IPV**  CA inc. the odds of past year IPV, aOR = 1.79 [1.31, 2.44]^b^*  CA inc. the odds of past year phy IPV, aOR = 1.76 [1.23, 2.53]^b^*  CSA inc. the odds of past year IPV, aOR = 2.05 [1.21, 3.48]^b^*  CSA inc. the odds of past year phy IPV, aOR = 2.14 [1.16, 3.92]^b^*  CPA & CSA inc. the odds of past year IPV, aOR = 3.00 [2.07, 4.35]^b^*  CPA & CSA inc. the odds of past year phy IPV, aOR = 2.73 [1.77, 4.20]^b^*  CPA & CSA inc. the odds of past year sexual IPV, aOR = 4.24 [1.77, 10.17]^b^*  CPA & CSA inc. the odds of past year phy & sexual IPV, aOR = 3.33 [1.60, 6.89]^b^*  **Revictimization**  CPA inc. the odds of LIPV phy revictimization, aOR = 1.62 [1.18, 2.22]^b^*  CSA inc. the odds of LIPV phy revictimization, aOR = 1.83 [1.10, 3.04]^b^*  CSA inc. the odds of LIPV sexual revictimization, aOR = 3.44 [1.64, 7.22]^b^*  CPA & CSA inc. the odds of LIPV phy revictimization, aOR = 2.69 [1.89, 3.84]^b^*  CPA & CSA inc. the odds of LIPV sexual revictimization, aOR = 4.82,[2.62, 8.87]^b^*  CPA & CSA inc. the odds of LIPV phy & sexual revictimization, aOR = 6.88 [4.03, 11.76]^b^*  **Poor past year health**  Any CA inc. the odds of poor past year health, aOR = 1.63 [1.26, 2.11]^b^*  CPA inc. the odds of poor past year health, aOR = 1.36 [1.02, 1.81]^b^*  CSA inc. the odds of poor past year health, aOR = 1.69 [1.08, 2.65]^b^*  CSA & CPA inc. the odds of poor past year health, aOR = 2.12 [1.56, 2.89]^b^*  CSA & CPA inc. the odds of poor past year health, aOR = 2.02 [1.42, 2.87]^ab^*  **Poor preg health**  Any CA inc. the odds of poor preg health, aOR = 1.32 [1.04, 1.68]^b^*  CSA & CPA inc. the odds of poor preg health, aOR = 1.88 [1.36, 2.59]^b^*  **Dep during preg**  CA inc. the odds of dep, aOR = 2.07 [1.58, 2.71]^b^*  CPA inc. the odds of dep, aOR = 2.05 [1.53, 2.75]^b^*  CPA & CSA inc. the odds of dep, aOR = 2.47 [1.79, 3.40]^b^*  CPA & CSA inc. the odds of dep, aOR = 2.14 [1.47, 3.11]^ab*^ | NA | |
| Belete (2020) | Postpartum dep | demos, alcohol use | Log. Reg | CSA inc. the odds of PPD, aOR = 2.8 [1.9, 4.27] **** | NA | |
| Benedict (1999) | Dep & preg outcomes (PTB, birthweight) | past/current abuse, stressors, substance use | Log. Reg | No significant findings for preg outcomes  CSA (contact or non-contact) & depressive symptoms (cutoff < 16), *ns*  CSA (contact or non-contact) inc. the odds of severe depressive symptoms (cutoff < 30), aOR = 2.44 [1.12, 5.31] | NA | |
| Bert (2009) | Parenting knowledge and behavior | a = type of mother  b = high-resource mothers | Multiple Reg. | **Responsiveness/empathy**  CPA predicts less responsiveness/empathy, β = -.16 **  CPA predicts less responsiveness/empathy, β = -.25 ^b^*  CEA predicts less responsiveness/empathy, β = -.12 ^a^*  **Attitudes towards punishment**  CPA predicts harsher attitudes towards punishment, β = .14 **  CPA predicts attitudes towards punishment, β = .26 ^b^*  CEA predicts harsher attitudes towards punishment, β = .15 ^a^**  **Attitudes towards child abuse/neglect**  CPA predicts less positive attitudes towards child abuse/neglect, β = .14 *  CEA predicts less positive attitudes towards child abuse/neglect, β = .15 ^a^**  **Child abuse potential**  CPA predicts greater child abuse potential, β = .41 ^b^**  CEA predicts greater child abuse potential, β = .15 ^a^**  **Authoritarian parenting**  CPA predicts greater authoritarian parenting, β = .13 * | *n/a* | |
| Blalock (2011) | Smoking | race | Log. Reg. | CPA inc. the odds of smoking 5 minutes after awake, aOR = 2.25 (1.04, 4.87]*  CEA inc. the odds of smoking 5 minutes after awake, aOR = 2.69 [1.32, 5.47]** | low or no trauma | |
| Brunst (2017) | Offspring asthma | demos, race, birth weight, gest age | Log. Reg. | no significant results for early-life abuse | NA | |
| Brunton (2020) | Pregnancy-related anxiety | age | Linear Reg. | CPA freq. predicts PrA (direct effect, β = 0.88***), resilience & social support partial mediators (adj effect, β = 0.42***)  CPY freq. predicts PrA, (direct effect, β = 1.57***), resilience & social support partial mediators (adj effect, β = 0.79***)  CSA freq. predicts PrA (direct effect, β = 0.32**), resilience & social support full mediators (adj effect, β = 0.04,*ns*) | *n/a* | |
| Bublitz (2014) | CAR & family functioning | no covariates were sig. associated, none included. | HLR modeling | CSA, not sig. associated with changes in prenatal CAR  CSA & poorer perceived family functioning predicted inc. in CAR across gest, β = .001, SE = .001** | *n/a* | |
| Bublitz (2012) | CAR | BMI & anxiety | ANOVA  HLR modeling | **Changes in CAR (nmol/L) *cf.* CSA group, CA (non-sexual abuse), and no abuse**  24 wks, *ns;* 30 wks, *ns;* 35 wks: *M_CSA_* = 6.58, *M_CA_* = 2.92 & *M_NA_* = 1.80 ** | Non-sexual abuse or NA | |
| Bublitz (2022) | NLR | Demos, prenatal BMI, prenatal smoking, & sleep-disordered breathing diagnosis. | Repeated measures ANOVA | CSA predicted change in NLR over preg (those with CSA had greater change) *F* = 6.39* | *n/a* | |
| Cammack (2017) | Smoking, PTB (< 37 wks), very PTB (< 34 wks) | a = childhood SES & race | Log. Reg. | **Smoking**  CSA (non-parent) inc. the risk of preg smoking, aRR = 1.64 [1.12-2.42]^a^  CPA (carer) & 2+ dep symptoms inc. the risk of preg smoking, aRR = 1.64 [1.11, 2.42]  CSA (non-parent/phy) & 2+ dep symptoms inc. the risk of smoking, aRR = 1.71 [1.08, 2.71]  CSA (non-parent) & 2+ dep symptoms inc. the risk of smoking, aRR = 1.73 [1.07, 2.81]  **PTB**  CSA (non-parent) inc. the odds of very PTB, aOR = 1.89 [1.07, 3.32]  **Stratified by race (other race = than black, white, or hispanic)**  Other race – CSA (non-parent/phy) inc. the odds of PTB, aOR = 3.99 [1.15, 13.84]  Other race – CSA (non-parent/phy) inc. the odds of very PTB, aOR = 7.54 [1.24, 45.75] | NA | |
| Cammack (2011) | Bacterial vaginosis | a = demos, race, substance use, sexual debut age,  b= discrim, stress, demos | Log. Reg. | CSA inc. the odds of persistent BV, aOR = 2.75 [1.00, 7.58]*^a^  CSA inc. the odds of persistent BV, aOR = 3.07 [1.09, 8.65]*^b^  CSA inc. the odds of persistent BV stratified by race: black people, OR = 1.65 nonblack people, OR = 2.93*^a^ | NA | |
| Castro (2003) | IPV in preg | SES & demos | Log Binomial Model | Moderate-high CPA assoc. with increased likelihood of preg IPV, aPR = 2.33 [1.70, 3.19]  Moderate-high CEA assoc. with increased likelihood of preg IPV, aPR = 2.75 [2.14, 3.53]  When other abuses were controlled, the above results were *ns* | NA or low abuse | |
| Choi (2018) | PPD dep, offspring maltreatment, maternal-infant bonding (1yr), child emotional/behavioral development & phy growth (1yr) | a = antenatal distress, age, socioeconomic disadvantage  b = other abuse types | SEM | CSA predicted higher PPD scores, β = .12^ab^**  CEA predicted higher PPD scores, β = .24^ab^***  CEA predicted higher child harm exp, through indirect pathways (direct effect CEA & maternal PPD, β = .24***, Maternal PPD & later maternal PPD, β = .63***, later maternal PPD & child harm exp. β = .18*; indirect effect β = .05**)  No significant results for CA or any subtype and bonding, development, or growth | *n/a* | |
| Chung (2008) | Prenatal dep | a = other ACEs,  b = demos | Log. Reg. | CPA inc. odds of dep, aOR = 1.35 [1.01, 1.81]^a^*  CSA inc. odds of dep, aOR = 1.77 [1.22, 2.57]^a^*  CSA inc. odds of dep, aOR = 1.69 [1.15, 2.48]^ab^*  CSA without positive maternal relationship inc. odds of dep, aOR = 3.20 | NA | |
| Cohen (2002) | PPD | Demos, mental health, psychosocial variables | Log. Reg. | no significant findings | NA | |
| Coles (2016) | Breastfeeding duration | a = smoking, b = adult violence, c = SES factors (e.g., partner status, age, income) | Log. Reg. | CSA inc. odds of breastfeeding ≥6 mths, OR = 0.78 [0.65, 0.93]*  CSA inc. odds of breastfeeding ≥6 mths, aOR = 0.81 [0.68, 0.98]^a^*  CSA inc. odds of breastfeeding ≥6 mths, aOR = 0.80 [0.67, 0.96]^b^*  CSA inc. odds of breastfeeding ≥6 mths, aOR = 0.89 [0.74, 1.07]^c^  CSA inc. odds of breastfeeding ≥6 mths, aOR = 0.90 [0.75, 1.09]^abc^ | No CSA | |
| Corona (2022) | Antenatal dep | demos, recruitment site, & parity. | Log. Reg. | CA inc. odds of dep, aOR = 2.01 [1.41, 2.86]  **Analyses of foreign-born and US-born Hispanic Latinas**:  Foreign-born, CA inc. odds of dep, aOR = 1.96 [1.16, 3.31]  US-born, CA inc. odds of dep, aOR = 2.40 [1.25, 4.60] | NA | |
| Cowell (2020) | PTB | Socio-demos & lifestyle characteristics | Poisson Reg. | No significant results for any abuse and PTB | *n/a* | |
| Diestel (2022) | PTSD symptoms |  | Network analyses | Examining CA and PTSD symptoms. Strength centrality, a measure of the association b/w variables, reported: CPA = 0.95, CSA = 0.59, CEA = 1.09 | *n/a* | |
| Dietz et al. (1999) | Unintended preg | Sociodemographics, age at first preg, sexual debut | Log. Reg. | Infrequent CPY inc. the risk of unintended preg, aRR = 1.1 [0.9, 1.3]*  Frequent CPY inc. the risk of unintended preg, aRR = 1.4 [1.2, 1.6]*  Infrequent CPA inc. the risk of unintended preg, aRR = 1.2 [1.0, 1.4]*  Frequent CPA inc. the risk of unintended preg, aRR = 1.5 [1.2, 1.8]*  Frequent CSA inc. the risk of unintended preg, aRR = 1.2 [1.1, 1.4]*  Peer sexual assault inc. the risk of unintended preg, aRR = 1.2 [1.0, 1.5]*  4+ types of abuse inc. the risk of unintended preg, aRR = 1.5 [1.2, 1.8]* | NA | |
| Drevin (2019) | Reproductive health & unplanned preg | Demos, birth country & other abuse | Chi-square  Poisson Reg | **Reproductive health**  Women reporting CPA were more likely to have sacral pain than ref, 72% *cf.* no CPA 35%***  Women reporting CPA were more likely to have pelvic pain than ref, 76% *cf.* no CPA 52%*  **Unplanned preg**  CEA/CPA inc. the risk of unplanned preg, aRR = 1.22 [1.14, 1.32]  CEA inc. the risk of unplanned preg, aRR 1.18 [1.13, 1.23]  CPA inc. the risk of unplanned preg, aRR 1.19 [1.07, 1.31]  CSA/CEA inc. the risk of unplanned preg, aRR 1.38 [1.27, 1.49]  CEA inc. the risk of unplanned preg, aRR = 1.17 [1.12, 1.23]  CSA inc. the risk of unplanned preg, aRR= 1.25 [1.17, 1.35]  CPA/CSA inc. the risk of unplanned preg, aRR = 1.29 [1.20, 1.39]  CPA inc. the risk of unplanned preg, aRR = 1.14 [1.05, 1.24]  CSA inc. the risk of unplanned preg, aRR = 1.26 [1.18, 1.34]  1 x instance of CA inc. the risk of unplanned preg, aRR 1.21 [1.16, 1.26]  2 x instances of CAs inc. the risk of unplanned preg, aRR 1.34 [1.25, 1.42]  3 x instances of CAs inc. the risk of unplanned preg, aRR 1.47 [1.36, 1.60] | NA | |
| Eide (2010) | Strong worries about the baby | demos  #multiparous women only | Log. Reg. | CPA inc. the odds of strong worries, OR = 1.62 [1.26, 2.08]  CSA inc. the odds of strong worries, aOR = 1.30 [1.03, 1.64]  CPA/CSA inc. the odds of strong worries, aOR = 2.10 [1.68, 2.62]  CPA/CSA inc. the odds of strong worries, aOR = 2.19 [1.46, 3.26]# | NA | |
| Elfgen (2017) | Breastfeeding complications | Yes, but not clearly stated | Log. Reg. | CSA inc. the odds of mastitis, OR = 2.84 [1.55, 6.45]*  CSA inc. the odds of painful breastfeeding, OR = 5.77 [1.55, 20.73]* | NA | |
| Farre-Sender (2018) | Mother-infant bonding disturbances | Reproductive & psychological variables | Lin. Reg. | CEA predicts less mother-infant bonding, β = 0.13, [0.02, 1.81]* | *n/a* | |
| Finy & Christian (2018) | Inflammation during preg (C-reactive protein) | race, gest, age, preg complications | SEM | CA predicts maternal serum C-reactive protein, β=0.16*  BMI mediates r’ship b/w CA & C-reactive protein BMI (direct effect: β =.20*; indirect effect: β =.11, SE= .04 [.02, .19]*) | *n/a* | |
| Freedman et al. (2017) | Risk of stillbirth | Sociodemographics | Log. Reg. | no significant results | No/ minimal abuse | |
| Galbally (2019) | Perinatal dep & parenting stress | Demos | Log. Reg. | moderate-severe CEA inc. the risk of dep diagnosis, RR = 1.81 [1.44-2.87]*  moderate-severe CPA inc. the risk of dep diagnosis, RR = 2.53 [1.31-4.87]**  moderate-severe CSA inc. the risk of dep diagnosis, RR = 4.22 [2.07-8.61]*** | None to mild abuse | |
| Gelaye (2016) | Migraine | a = age, ethnicity, b = lifetime IPV | Log. Reg. | CA inc. the odds of migraine, aOR = 1.38 [1.16, 1.64]^a^*  CA inc. the odds of migraine, aOR = 1.28 [1.08, 1.53]^ab^*  CPA inc. the odds of migraine, aOR = 1.22 [1.01, 1.48]^a^*  CPA/CSA inc. the odds of migraine, aOR = 1.71 [1.34, 2.10]^a^*  CPA/CSA inc. the odds of migraine, aOR = 1.55 [1.24, 1.92]^b^*  6+ instances of CA inc. the odds of migraine, aOR = 2.38 [1.68, 3.43]^a^*  6+ instances of CA inc. the odds of migraine, aOR = 2.14 [1.50, 3.06]^ab^* | NA | |
| Gelaye (2015) | Sleep quality | a = age & race, b = IPV 12 mths prior to preg | Log. Reg. | **Stress-related sleep disturbance (SRSD)**  CA (any CPA or CSA) inc. the odds of SRSD, aOR = 1.65 [1.15, 2.38]^a^*  CA inc. the odds of SRSD, aOR = 1.65 [1.15, 2.38]^ab^*  CPA/CSA inc. the odds of SRSD, aOR = 2.26 [1.44, 3.53] ^a^*  CPA/CSA inc. the odds of SRSD, aOR = 2.10 [1.33, 3.30] ^ab^*  3+ occurrences CA inc. the odds of SRSD, OR = 2.35 [1.57, 3.51]^a^*  CA (mediated by IPV 12 mths prior to preg & dep) inc. the odds of SRSD, aOR = 1.67 ^a^*  **Poor sleep quality (PSQ)**  CA inc. the odds of PSQ, aOR = 2.11 [1.35, 3.30]^a^*  CA inc. the odds of PSQ, aOR = 1.98 [1.26, 3.12]^a^*  CPA inc. the odds of PSQ, aOR = 1.95 [1.20, 3.16]^a^*  CPA inc. the odds of PSQ, aOR = 1.92 [1.18, 3.13]^ab^*  CPA/CSA inc. the odds of PSQ, aOR = 2.43 [1.45, 4.09]^a^*  CPA/CSA inc. the odds of PSQ, aOR = 2.18 [1.29, 3.69]^ab^*  3+ occurrences CA inc. the odds of PSQ, OR = 2.57 [1.59, 4.13]^a^*  CA (mediated by IPV 12 mths prior to preg & dep) inc. the odds of PSQ, aOR = 2.18 ^a^* | NA | |
| Giallo (2017) | Perinatal depressive symptoms | a = Sociodemographic, preconception, antenatal & postnatal factors | Multivariable Log. Reg. using classes determined by longitudinal latent class modeling. | **Subclinical dep**  CPA inc. the odds of subclinical dep, OR = 2.30 1.75, 3.04]***  CPA inc. the odds of subclinical dep, aOR = 1.81 [1.32, 2.48]^a^***  CSA inc. the odds of subclinical dep, OR =1.69 [1.25, 2.29]****  **Persistently high dep**  CPA inc. the odds of persistently high dep, OR = 5.01 [3.19, 7.88]***  CPA inc. the odds of persistently high dep, aOR = 2.82 [1.63, 4.87]^a^***  CSA inc. the odds of persistently high dep, OR = 2.20 [1.35, 5.59]** | NA | |
| Heimstad (2006) | Complicated delivery (i.e., operative vaginal delivery (forceps or vacuum) or CS (planned and emergency); | not stated | Log. Reg | CSA inc. the odds of a complicated delivery, OR = 2.5 [1.5, 4.4]  CPA inc. the odds of a complicated delivery, OR = 2.3 [1.4, 3.9] | NA | |
| Huth-Bocks (2013) | Post-Traumatic Stress | age & income | Lin. Reg. | No significant results for individual abuse types | *n/a* | |
| Hyle (1995) | PTB and low birth weight | Parity, age, marital status, attachment, gest age | Lin. Reg. | CSA (broad def) predicts birth weight, β = .11*  CSA intrafamilial predicts birth weight, β = .12*  CSA extrafamilial predicts birth weight, β = .10*  CSA extrafamilial (3^rd^ incident) predicts birth weight, β = -.11*  Age of onset of CSA predicts birth weight, β = .12*  Completed versus attempted assault predicts birth weight, β = .10*  CSA degree of upset (14-17 years) predicts birth weight, β = .09*  Non-disclosure of CSA predicts birth weight, β = .09^a^*  CSA 3 incidences < 14 (narrow def), offender present predicts birth weight, β = .12*  CSA 3 incidences < 14 predicts gest age, β = - .16^a^* | *n/a* | |
| Jantzen (1998) | Cocaine use and adult victimization | Not stated | Chi-Square | CA (CPA or CSA) more often reported lifetime cocaine use than ref, 41% v 17% ***  CA (CPA or CSA) more often reported perinatal cocaine use than ref, 14% v 6% ***  CSA more often reported lifetime cocaine use 53% than ref ****  CSA/CPA more often reported lifetime cocaine use 55% than ref ****  CPA more often reported lifetime cocaine use, 26 % than ref ****  More severe abuse (i.e., sexual penetration or severe beating) was associated with lifetime cocaine use (47%) than less severe abuse (30% )*  CA (CPA or CSA) more often reported phy or sexual abuse during preg, 13% v 5% ** | NA | |
| Kang (2022) | Dep | timing of EPDS measurement, demos, disability, single parent status, multicultural status, psychosocial & lifestyle factors | Log. Reg. | CA & peripartum dep, aOR = 1.91 [1.74, 2.11] | NA | |
| Khanlari (2019) | PND | antenatal alcohol, smoking, infant’s sex, BMI, birth facility. |  | CA inc. the odds of antenatal distress, aOR = 1.9 [1.61–2.32]***  CA inc. the odds of high antenatal dep, aOR = 3.2 [2.6, 3.8]***  CA inc. the odds of postnatal distress, aOR = 1.4 [1.07, 1.80]*  CA inc. the odds of high postnatal dep, aOR = 1.9 [1.4, 2.5]*** | NA | |
| Kiewa (2022) | PND | age and number of births | Log. Reg. | A prior history of dep & CEA inc. the odds of PND, OR = 1.4 [1.1, 1.7] |  | |
| Kunseler (2016) | Parenting self-efficacy | Demos |  | CA predicts less parenting self-efficacy after exp to difficult infant, β = -4.32, SE = 2.13 * | *n/a* | |
| Leeners (2016) | Childbirth experiences | Psychosocial & childbirth related variables | Chi-square  Log Reg. | **Women with a CSA history were less likely to:**   - participate in childbirth classes, 72.9% v 84.1%* - participate in childbirth classes alone, 62.4% v 75.9%* - participate in childbirth classes with their partner, 36.3% v 61.2%** - feel prepared for labor, 62.4% v 75.9%* - have a trusted person with them during labor, 75.3% v 86.5%* - deliver spontaneously, 78.8% v 90.0%* - have pain during childbirth, 69.4% v 82.9%*** - have no pain relief during childbirth, 49.4% v 65.3%* - participate in medical decisions, 60.0% v 87.1%**   **Women with a CSA history were more likely to have:**   - an intense fear of delivery, 24.7% v 5.3%** - general anesthesia during childbirth, 14.1% v 7.6%* - a preterm birth, 18.8% v 8.2%* - an extreme duration of delivery, 31.8% v 11.2%** - a major difficulty with vaginal exams, 54.1% v 27.6%*** - difficulty with male staff, 55.3% v 18.8%*** - difficulty with nakedness, 58.8% v 28.2%***   CSA inc. the odds of a negative labor perception, OR = 2.12 [1.27, 3.53]**  CSA inc. the odds of fear of delivery, OR = 2.73 [1.40, 5.30]** | No CSA | |
| Leeners (2010) | Preg complications | other ACEs | Chi-square  Log. Reg. | **Women with a CSA history were more likely to have**:   - premature contractions, 38.8% v 20.0%** - cervical insufficiency, 25.9% v 9.4%*** - hospitalization during preg, 41.2% v 19.4%**** - premature birth (< 37 wks), 18.8% v 8.2%*   CSA inc. the odds of preg complications, aOR = 1.4 [1.14, 1.94]**  CPA inc. the odds of preg complications, aOR = 1.2 [0.64, 1.56]* | NA | |
| Leeners (2013) | Prenatal care | a = demos, dissociation, gest age, other ACEs, preg complications | Chi-square  Log. Reg. | **Women with a CSA history were more likely to have**:   - a baby with a birth weight (< 2800 grams), 20.2% v 13.4%* - a baby with a birth weight (> 3500 grams), 48.0% v 33.6%* - a reduced number of prenatal consultations, aOR = 0.214 [.054, .56]**   **Women with a CSA history were less likely to have:**   - a baby with a birth weight ≥2800 to < 3500 grams), 31.6% v 53.0%*   CSA exp. triggers for memories during preg, inc. odds of reduced consultations, aOR = 1.34 [1.09, 1.76]**  CSA with preg phy abuse, inc. odds of reduced consultations, aOR = 0.30 [0.06, 0.95]* | No CSA | |
| Lehnig (2019) | Mother-infant bonding | Postpartum mental health | HLR | No significant results | *n/a* | |
| Lev-Wiesel (2009) | Childbirth as retraumatizing event & PTSD | a = prenatal PTSD, b = disassociation tendency  c = demos, d = prenatal avoidance e = prenatal arousal | Multiple Lin. Reg | **Regression analyses**  CSA predicts greater PTSD 7 mths after childbirth PP, β= .12^abc^**  CSA predicts greater dissociation tendency, 2 mths after childbirth, β= .18 ^ac^**  CSA predicts greater avoidance 7 mths after childbirth, β= .10 ^cd^**  CSA predicts greater arousal levels 7 mths after childbirth, β= .11^ce^** | no or other-trauma | |
| Li (2017) | Dep | Sociodemographics, stress, unintended preg, parity, preg complications | GLM  Log. Reg. | CEA inc. the odds of antepartum dep, aOR = 4.26 [1.24, 14.64]*  CSA inc. the odds of antepartum dep, aOR = 5.05 [1.27, 20.00]*  CEA interacts with time in predicting antepartum dep, β = -1.23 [-2.38, -0.07]* (impact of CEA on dep lessens over time to levels below the ref group [i.e., no CEA]). | NA for the grp compared | |
| Littleton (2015) | Somatic complaints during preg and dep symptoms | Demos, planned preg, number of children in the home | Lin. Reg. | Dep. symptoms mediates the r’ship b/w CSA & somatic complaints, (indirect path β = 1.30 [-0.74, 2.50], *p* = .12) | *n/a* | |
| Lukasse (2015) | Unintended preg | Demos, gest age |  | CA inc. the odds of unintended preg, aOR = 1.49 [1.28,1.73]  CPA inc. the odds of unintended preg, aOR = 1.55 [1.27, 1.89]  CSA inc. the odds of unintended preg, aOR = 1.66 [1.37, 20.2]  CEA inc. the odds of unintended preg, aOR = 1.55 [1.28, 1.86] | NA | |
| Lukasse (2009) | Common complaints in preg | a = Sociodemographics & other preg risk factors, b = adult abuse & mental distress |  | Any CA inc. the odds of 7+ common complaints, aOR = 2.1 [1.9, 2.2]^a^  CA inc. the odds of 7+ common complaints, aOR = 3.1 [2.6, 3.5]^ab^  CEA inc. the odds of 7+ common complaints, aOR = 1.6 [1.5,1.7]^ab^  CPA inc. the odds of 7+ common complaints, aOR = 1.6 [1.4, 1.9]^ab^  CSA inc. the odds of 7+ common complaints, aOR = 1.7 [1.5,1.9]^ab^  CEA/CPA inc. the odds of 7+ common complaints, aOR = 2.3 [2.0, 2.6]^ab^  CEA/CSA inc. the odds of 7+ common complaints, aOR = 2.4 [2.0, 2.8]^ab^  CPA/CSA inc. the odds of 7+ common complaints, aOR = 2.2 [1.8, 2.7]^ab^ | NA | |
| Lukasse (2010a) | Fear of Childbirth | a = Demos, planned preg, adult abuse, dep, prev negative birth experience | Log. Reg. | **Primiparous only**  CSA inc. the odds of severe FoC, OR = 1.91 [1.12, 3.27]*  Severe CSA inc. the odds of severe FoC, OR = 3.45 [1.66, 7.17]*  CPA inc. the odds of severe FoC, OR = 3.06 [1.91, 4.90]*  Severe CPA inc. the odds of severe FoC, OR = 2.36 [1.00, 5.60]*  CEA inc. the odds of severe FoC, OR = 3.49 [2.17, 5.49]*  Severe CEA inc. the odds of FoC, OR = 2.95 [1.51, 5.76]*  Any CA inc. the odds of FoC, OR = 2.48 [1.70, 3.64]*  CPA/CSA inc. the odds of severe FoC, OR = 3.15 [1.42, 6.95]*  CSA/CEA inc. the odds of severe FoC, OR = 4.04 [1.97, 8.30]*  CPA/CEA inc. the odds of severe FoC, OR = 3.96 [2.14, 7.33]*  CPA/CSA/CEA inc. the odds of severe FoC, OR = 5.30 [2.03, 13.86]*  Any CA inc. the odds of severe FoC, aOR = 2.00 [1.30, 3.08]^a^*  **Multiparous only**  Non-contact CSA inc. the odds of severe FoC, OR = 1.80 [1.10, 3.00]*  Mild CSA (humiliation) inc. the odds of severe FoC, OR = 2.35 [1.05, 5.27]*  CPA inc. the odds of severe FoC, OR = 1.78 [1.10, 2.91]*  Severe CPA inc. the odds of severe FoC, OR = 2.67 [1.28, 5.58]*  CEA inc. the odds of severe FoC, OR = 1.78 [1.10, 2.91]*  Any CA inc. the odds of severe FoC, OR = 1.50 [1.02, 2.16]*  Any CA inc. the odds of severe FoC, aOR = 1.17 [0.76, 1.80]^a^*  Any CA inc. the odds of positive birth exp. OR = 1.20 [0.90, 1.60]*  Any CA inc. the odds of negative birth exp. OR = 5.87 [3.19, 10.84]*  No CA inc. the odds of negative birth exp. OR = 8.95 [5.72, 14.01]* | NA | |
| Lukasse (2010b) | Cesarean section | a = Demos, adult abuse, preg risk factors | Multivariate Log. Reg. | Any CA inc. the odds of a CS during labor, OR = 1.16 [1.05, 1.28]  Any CA inc. the odds of a CS during labor, aOR = 1.16 [1.03, 1.30]^a^  Mild CEA inc. the odds of a CS during labor, OR = 1.14 [1.01, 1.29] | NA | |
| Lukasse (2011) | Fear of Childbirth & Wish for Cesarean | a = Demos, BMI, adult abuse, mental distress,  b = FoC 1^st^ preg, prev mode of delivery & birth experience. | Log. Reg. | **Multiparous women and second preg**  Any CA inc. the odds of FoC, aOR = 1.31 [1.02, 1.66]^ab^  Mild CEA inc. the odds of FoC, aOR = 1.43 [1.09, 1.66]^a^  Severe CEA inc. the odds of FoC, aOR = 1.58 [1.02, 2.45]^a^  Any CEA inc. the odds of FoC aOR = 1.45 [1.11, 1.88]^a^  Any CA inc. the odds of Wish for CS, aOR = 1.54 [1.03, 2.31]^ab^  CPA inc. the odds of Wish for CS, aOR = 2.01 [1.07, 3.77]^ab^  CSA inc. the odds of Wish for CS, aOR = 2.14 [1.18, 3.89]^ab^ | NA | |
| Lydsdottir (2019) | CMDs in preg (i.e., dep, PTSD, anxiety disorders) | other ACEs | Log. Reg. | CPA inc. the odds of CMD, aOR = 5.17 [2.81, 9.51]***  CSA inc. the odds of CMD, aOR = 2.96 [1.92, 4.57]*** | No CMD | |
| Madigan (2017) | Prenatal risk factors (biomedical [e.g., diabetes, hypertension], psychosocial [e.g., income, education]) | age, immigration status | Log. & Poisson Reg. | CSA inc. the odds of biomedical risk, aOR = 1.66 [0.89, 3.09]*  CPA inc. the odds of biomedical risk, aOR = 2.04 [1.29, 3.22]**  CSA inc. the odds of psychosocial risk, aOR = 2.65 [1.33, 5.28]**  CPA inc. the odds of psychosocial risk, aOR = 2.97 [1.80, 4.89]*** | NA | |
| Mahenge (2018) | PPD | Demos, partner age, preg wantedness, other ACES | Multivariate Log. Reg. | CPA inc. the odds of PPD, aOR = 2.6 [1.50, 4.57]*  CSA inc. the odds of PPD, aOR = 2.7 [1.35, 5.41]*  CPY inc. the odds of PPD, aOR = 2.5 [1.44, 4.49]*  CSA/CPY inc. the odds of PPD, aOR = 3.8 [2.12, 6.87]*  Any CA inc. the odds of PPD, aOR = 5.2 [2.63, 10.50]*  Any CA (CPS, CSA, CPY)/any preg IPV inc. the odds of PPD, aOR = 9.1 [4.50, 18.59]* | NA | |
| Malta (2012) | Dep, anxiety, stress, & parenting morale. | Income, psychological & psychosocial variables, postpartum energy levels | Log. Reg. | No significant findings for anxiety or stress  CA inc. the odds of PND, aOR = 1.83 [1.11, 2.99]*  CA inc. the odds of low parenting morale, aOR = 1.78 [1.13, 2.81]* | NA | |
| Margerison-Zilko (2017) | Preterm birth | Demos, parity | Log. Reg. | CA inc. the odds of late PTB, aOR = 1.5 [1.0, 2.2]*  CSA inc. the odds of late PTB, aOR = 1.5 [1.0, 2.2]* | NA | |
| Mason (2016) | Gestational diabetes mellitus | a = Demos, age 5 body size, diabetes.  b = BMI > 25  c = not overweight at 18 years,  d = overweight at 18 years |  | Moderate CPA inc. the risk of GDM, aRR = 1.16 [1.04, 1.29]^a^  Severe CPA inc. the risk of GDM, aRR = 1.42 [1.21, 1.66]^a^ Severe CSA inc. the risk of GDM, aRR = 1.30 [1.14, 1.49]^a^ Severe CPA/any CSA inc. the risk of GDM, aOR = 1.68 [1.38, 2.04]^a^  Severe CSA/any CPA inc. the risk of GDM, aRR = 1.42 [1.20, 1.67]^a^  Moderate CPA inc. the risk of GDM, aRR = 1.09 [1.04, 1.29]^ab^  Severe CPA inc. the risk of GDM, aRR = 1.43 [1.22, 1.67]^ab^  Severe CSA inc. the risk of GDM, aRR = 1.31 [1.15, 1.50]^ab^  Severe CPA inc. the risk of GDM, aRR = 1.43 [1.22, 1.67]^ac^  Severe CSA inc. the risk of GDM, aRR = 1.30 [1.12, 1.50]^ac^  Mild CPA inc. the risk of GDM, aRR = 1.52 [1.04, 2.21]^ad^  Moderate CPA inc. the risk of GDM, aRR = 1.74 [1.26, 2.40]^ad^  Severe CPA inc. the risk of GDM, aRR = 1.74 [1.09, 2.78]^ad^  Moderate CSA inc. the risk of GDM, aRR = 1.40 [1.03, 1.90]^ad^  CSA (touch only) inc. the risk of GDM, aRR = 1.11 [1.00, 1.23]^a^  CPA in adolescence inc. the risk of GDM, aRR = 1.36 [1.12, 1.64]^a^  CPA in adolescence inc. the risk of GDM, aRR = 1.37 [1.12, 1.66]^ab^  CSA in childhood inc. the risk of GDM, aRR = 1.29 [1.14, 1.46]^a^  CSA in childhood inc. the risk of GDM, aRR = 1.24 [1.09, 1.41]^ab^ | NA | |
| Mayhew (2022) | Postanal anxiety |  | HLR | Reg. model included prenatal anxiety and distressing childbirth.  CEA predicts postnatal anxiety, β = .12*** | *n/a* | |
| McNaughton Reyes (2020) | IPV | Demos, prev preg | GLM | No significant main findings for IPV exp | *n/a* | |
| Mitro (2019) | Placental abruption | Demos & parity | Log. Reg. | 3 + CA inc. odds of PA, aOR = 1.56 [1.11, 2.19]**  Severe CA/IPV inc. odds of PA, aOR = 2.06 [1.25, 3.40]**  Early PTB deliveries & 3+ CA inc. odds of PA, aOR = 1.87 [1.22, 2.87]**  Full-term deliveries & severe CA/IPV inc. odds of PA, aOR = 2.85 [1.56, 5.20)** | No or rare abuse | |
| Nagl (2017) | Postpartum mental health | a = demos, b = co-occurrence of different types of maltreatment. | Log. Reg. | Severe CSA inc. odds of dep, aOR = 9.46 [3.62, 24.69]^a^*  Severe CSA inc. odds of dep, aOR = 3.81 [ 1.28, 11.30]^ab^*  Slight CPA inc. odds of dep, aOR = 6.24 [2.45, 15.89]^a^*  Slight CPA inc. odds of dep, aOR = 3.43 [ 1.21, 9.77]^ab^*  Moderate CPA inc. odds of dep, aOR = 14.93 [4.75,46.94]^a^*  Moderate CPA inc. odds of dep, aOR = 5.56 [1.52, 20.42]^ab^*  Severe CPA inc. odds of dep, aOR = 8.63 [2.49, 29.90]^a^*  Slight CEA inc. odds of dep, aOR = 3.13 [1.33,7.39]^a^*  Moderate CEA inc. odds of dep, aOR = 9.32 [3.03, 28.67]^a^*  Moderate CEA inc. odds of dep, aOR = 4.96 [1.30, 18.99]^ab^*  Severe CEA inc. odds of dep, aOR = 17.36 [7.47,40.38]^a^*  Severe CEA inc. odds of dep, aOR = 8.28 [ 2.28, 30.04]^ab^*  Severe CSA inc. odds of anxiety, aOR = 6.72 [2.75, 16.41]^a^*  Severe CSA inc. odds of anxiety, aOR = 3.70 [ 1.42, 9.61]^ab^*  Slight CPA inc. odds of anxiety, aOR = 2.63 [ 1.23, 5.65]^a^*  Moderate CPA inc. odds of anxiety, aOR = 4.15 [1.46, 11.79]^a^*  Severe CPA inc. odds of anxiety, aOR = 3.63 [1.22, 10.83]^a^*  Slight CEA inc. odds of anxiety, aOR = 3.09 [1.85, 5.16]^a^*  Slight CEA inc. odds of anxiety, aOR = 2.46 [1.42, 4.30]^ab^*  Moderate CEA inc. odds of anxiety, aOR = 7.65 [ 2.97, 19.70]^a^*  Moderate CEA inc. odds of anxiety, aOR = 5.12 [ 1.81, 14.46]*^b^  Severe CEA inc. odds of anxiety, aOR = 6.23 [2.99,12.97]^a^*  Severe CEA inc. odds of anxiety, aOR = 3.81 [1.43, 10.16]^b^* | NA | |
| Nerum (2013) | Labor outcomes | Demos, BMI, preg history, obstetric risks | Chi-square  Log. Reg | No sig. results for CSA and operative delivery or CS  **Women with a CSA history were more likely to (***cf. to controls***):**   - smoke, 38% v 20%** - be single and unsupported, 35% v 8%** - be unemployed, 39% v 8%** - have a prev termination or miscarriage, 34% v 23%** - have more obstetric risks, 46% v 22%** - have labor induced/ augmented with oxytocin, 57% v 55%** - have epidural analgesia, 42% v 31%** - be transferred to neonatal intensive care, 13% v 4%** | Adult rape & NA | |
| Nieto (2017) | Maternal attachment | Dep, adjustment, social support | Log. Reg. | CSA inc. odds of less maternal attachment, aOR = 2.77 [1.17, 6.55]* | *n/s* | |
| Ogbo (2019) | Distress and depressive symptoms | Birthing facility, baby’s gender, psychosocial & obstetric factors | Log. Reg. | CA inc. odds of antenatal distress, aOR = 1.9 [1.4, 2.6] ***  CA inc. odds of antenatal dep symptoms, aOR = 3.2 [2.4, 4.3] ***  CA inc. odds of postnatal dep symptoms, aOR = 2.0 [1.2, 3.1]** | NA | |
| Oliveira (2017) | PTSD | Demos, psychological & obstetric history, other traumas, alcohol use. | Path analysis | CSA direct pathway to PTSD, *p* < .001. CSA assoc. with PTSD symptoms through a sequence of mediators (traumatic event, psychiatric history, psych IPV during preg, FoC) and indirect pathway from CSA to PTSD, *p* = .066 | *n/a* | |
| Plaza (2012) | Thyroid status and postnatal depressive symptomology | a = other abuses, prev affective disorder, income | Log. Reg. | CPA inc. odds of PPD, OR = 5.0 [2.2-11.6]*  CEA inc. odds of PPD, OR = 2.7 [1.1-6.4]*  CSA inc. odds of PPD, OR = 2.6 [1.1-6.0]*  CPA inc. odds of thyroid dysfunction, aOR = 3.95 [1.23, 12.71]*  CPA inc. odds of PPD, aOR = 5.5 [2.2–13.7]^a^**** | NA | |
| Racine (2018) | Reproductive risk (prev stillbirth), preg psychosocial difficulties (support), & complications (hypertension) | Demos, parity | Log. Reg | CPA/CEA inc. odds of pre-preg health risk, aOR = 1.25 [1.02, 1.54]*  CPA/CEA inc. odds of psychosocial difficulties in preg, aOR = 1.60 [1.34, 1.89] ***  No sig. results for CSA | NA | |
| Racine (2020) | Antenatal substance use | a = Demos | Multivariate Log. Reg. | CSA inc. the odds of drug use, aOR = 1.68 [1.0, 2.83]^a^  Family violence (CPA, CEA & exp to DV) inc. the odds of binge drinking OR = 1.81 [1.43, 2.28]  Family violence inc. the odds of binge drinking, aOR = 1.65 [1.29, 2.10]^a^  Family violence inc. the odds of drug use, OR = 1.87 [1.29, 2.70]  Family violence inc. the odds of drug use, aOR = 1.50 [1.01, 2.23]^a^  Family violence inc. the odds of smoking, OR = 2.37 [1.88, 3.00]  Family violence inc. the odds of smoking, aOR = 2.13 [1.65, 2.73]^a^ | NA | |
| Ranchod (2016) | Preg weight gain | Demos, childhood environment, foreign born | Log Binominal models | 2+ instances CPA inc. risk of excess gestational weight gain, aRR = 1.2 [1.1-1.4] * | NA | |
| Rich-Edwards (2011) | Prenatal dep | age and race | Log. Reg. | CA (CPA or CSA) inc. odds of dep, aOR = 1.26 [1.00, 1.59] | NA | |
| Roberts (2013) | Offspring autism | Maternal SES in childhood, demos, sex of the child | GEEs | Severe CSA inc. risk of autism, aRR = 2.0 [1.3, 3.1]**  Severe CPA/CEA inc. risk of autism, aRR = 1.6, [1.3, 2.1]***  CA 6+ occurrences inc. risk of autism, aRR = 3.0 [1.9, 4.8]***  Mediation results not fully reported | NA | |
| Robertson-Blackmore (2013) | Antenatal and PPD | Demos | Log. Reg. | CPA/neglect no significant findings for antenatal dep & PPD  CPA/neglect inc. odds of lifetime PTSD, aOR = 12.16 [3.96, 37.39]***  CSA inc. odds of antenatal dep, aOR = 2.47 [1.27, 4.78]**  CSA inc. odds of lifetime PTSD, aOR = 9.21 [4.85, 19.45]*** | No trauma | |
| Samia (2020) | Psychosocial distress in preg (PrA, dep, perceived stress) | Sociodemographic | REML | No significant findings for contact CSA & PrA and perceived stress  Contact CSA predicts antenatal dep, β = 2.24 (0.76) **  No time (12-19 & 22-29 wks) & CSA interaction for antenatal dep, β = -1.41 (0.88)*ns*. | *n/a* | |
| Sanchez (2017) | PTSD | a = age, ethnicity  b = lifetime experiences with IPV | Log. Reg | CA predicted PTSD (direct effect OR = 7.19), mediated by Lifetime IPV, adjusted direct effect aOR = = 5.73 [2.99, 10.98]^a b^  CPA inc. odds of PTSD, aOR = 4.31 [2.18, 8.49]^a^  CSA inc. odds of PTSD, aOR = 5.33 [ 2.38, 11.98]^a^  CPA/CSA inc. odds of PTSD, aOR = 8.03 [4.10, 15.74]^a^  CA inc. odds of PTSD, aOR = 5.13 [2.02, 12.97]^a^  CA/IPV inc. odds of PTSD, aOR = 20.20 [8.18, 49.85]^a^ | NA or lifetime IPV | |
| Schei (2014) | Operative delivery & cesarean section | a = age, twin preg, < 37 wks gest, country of residence, b = prev CS | Log. Reg. | **Multiparous women only**  CPA inc. odds of emergency CS, aOR = 1.51 (1.05, 2.19)^a^  CPA inc. odds of emergency CS, aOR 1.48 (1.01–2.18)^ab^ | NA | |
| Schreier (2015) | Cortisol | Demos, PTSD symptoms | ANOVA, Chi-square | Women who exp. CPA/CSA had higher hair cortisol levels than ref, *t*(166) = 2.65***  When stratified by race, the assoc. b/w CA and cortisol was sig. only for black women, F(2,23) = 5.37* *cf.* to White and Hispanic women. | NA | |
| Seng (2014) | PTSD | a = sociodemographic risk, other traumas,  b = PTSD symptoms & psychogenic amnesia | Cluster analysis & Log Reg. | CPA inc. odds of being in Affect/Relational Dysregulation cluster, aOR = 2.3 [1.2, 4.4]^a^*  CPA inc. odds of being in Healthy cluster, aOR = 0.48 [0.32, 0.72]^a^*  CSA inc. odds of being in Comorbid PTSD cluster, aOR = 2.4 [1.3, 4.4]^a^*  CPA inc. odds of being in Healthy cluster, aOR = 0.61 [0.38, 0.98]^ab^*  CSA inc. odds of being in Comorbid PTSD cluster, aOR = 1.9 [1.0, 3.7]^ab^* | All other clusters | |
| Senior (2005) | Eating disorder symptomology | a = Demos, other traumas, positive childhood experiences | Log. Reg. | **Lifetime ED**  CPA inc. odds of lifetime ED, OR = 2.53 [1.95, 3.29]***  CEA inc. odds of lifetime ED, OR = 2.12 [1.75, 2.56]***  CSA inc. odds of lifetime ED, OR = 2.16 [1.86, 2.51]***  CSA inc. odds of lifetime ED, aOR = 1.76 [1.49, 2.07]^a^***  **Antenatal ED**  CSA inc. odds of antenatal ED, OR = 2.04 [1.40-2.95] ***  CEA inc. odds of antenatal ED, OR = 2.17 [1.42-3.33]***  **Antenatal shape or weight concern**  CPA inc. odds of shape concern, OR = 1.58 [1.24, 2.00]***  CSA inc. odds of shape concern, OR = 1.56 [ 1.38, 1.76]***  CEA inc. odds of shape concern, OR = 1.59 [1.36, 1.87]***  CSA inc. odds of shape concern, aOR = 1.32 [1.16, 1.51]^a^***  CPA inc. odds of weight concern, OR = 1.78 [1.39, 2.29]***  CSA inc. odds of weight concern, OR = 1.74 [ 1.53, 1.98]***  CEA inc. odds of weight concern, OR = 1.69 [1.42, 2.00]***  CSA inc. odds of weight concern, aOR = 1.46 [1.27, 1.68]^a^*** | NA | |
| Shamblaw (2021) | Perinatal/obstetric complications | a = Sociodemographics;  b = various lifetime psychological disorders | Log. Reg. | CSA inc. odds of perinatal/obstetric complications aOR = 2.29 [1.43, 3.64]***^a^  CSA inc. odds of perinatal/obstetric complications, aOR = 2.00 [1.20, 3.32]**^ab^  CEA inc. odds of perinatal/obstetric complications, aOR = 2.19 [1.25, 3.84]***^a^ | NA | |
| Sorbo (2015) | Breastfeeding cessation | adult abuse, maternal age, education, and civil status | Log. Reg. | CA inc. odds of breastfeeding cessation ≤ 4 mths, aOR = 1.12 [1.05, 1.20]*  CSA inc. odds of breastfeeding cessation ≤ 4 mths, aOR = 1.22 [1.11, 1.65]*  CEA &/or CPA inc. odds of breastfeeding cessation ≤ 4 mths, aOR = 1.06 [0.98, 1.15]* | NA | |
| Stark Stigger (2020) | Maternal-fetal attachment | SES, demos, prenatal care; alcohol | Lin. Reg. | CEA predicts less maternal-fetal attachment, β = -0.2, [-0.5, -0.0]* | *n/a* | |
| Stephens (2021) | Diurnal cortisol rhythms during preg | proximal stressors | HLR models | CSA predicts CAR 30 minutes, β = 0.18, SE = 0.06**  CSA predicts CAR 60 minutes, β = 0.22, SE = 0.06*** | No or low-to-moderate CM | |
| Sumner (2012) | PTSD | Sociodemographics, dep symptoms, lifetime IPV, social support | Lin. Reg. | CA predicts PTSD during preg, β = .24** | *n/a* | |
| Swanson (2014) | PP sleep complaints | a = dep, PTSD diagnosis,  b = infant night wakening |  | CPA inc. odds of falling asleep, aOR = 9.20 [2.20, 38.45]^a^**  CPA inc. odds of staying asleep, aOR = 3.84 [1.04, 14.24]^ab^*  CPA/CSA inc. odds of falling asleep, aOR = 5.95 [1.50, 23.57]^a^**  CPA/CSA inc. odds of staying asleep, aOR = 3.56 [1.03, 12.31]^ab^* | NA | |
| Tebeka (2021) | PP dep | a = sociodemographic variables (i.e., age, marital status, education level & employment)  b = all other predictors in the model | Log. Reg. | CEA inc. odds of early-onset PPD (8-wks PP), aOR = 2.2 [1.1, 4.2]^a^*  CEA inc. odds of late-onset PPD (1-yr PP), aOR = 3.6 [2.0, 6.4]^a^***  CPA inc. odds of late-onset PPD, aOR = 2.8 [1.3, 5.6] ^a^**  CSA inc. odds of late-onset PPD, aOR = 2.7 [ 1.4,5.4] ^a^**  CSA inc. odds of early-onset PPD aOR = 2.0 [1.0, 4.0]^ab^  CEA inc. odds of late-onset PPD, aOR = 2.6 [1.4, 4.8]^ab^ | NA | |
| Yampolsky (2010) | PTS symptoms, dep, chronic illness & gynecological problems as contributing to high risk preg status | a = dep, PTS,  b = dep, intrusion, avoidance & arousal | Log. Reg. | CSA inc. odds of high dep symptoms, OR = 1.52***  CSA inc. odds of high PTS symptoms, OR = 2.06***  CSA inc. odds of high avoidance symptoms, OR = 1.60^a^**  CSA inc. odds of gynecological problems, aOR = 0.36^a^****  CSA inc. odds of gynecological problems, aOR = 1.94^b^*** | Other or NA | |
| Zhang (2020) | Suicide ideation | a = Demos, ethnicity, nulliparity, unplanned preg, history of abortion, and mental health disorders, b = dep, c = CA total score | Multivariate Log. Reg. | Any experience of CA (CPA, CSA, or CEA) inc. odds of suicide ideation, aOR = 3.11 [1.75, 5.53]^a^  Any experience of CA inc. odds of suicide ideation, aOR = 2.44 [1.31, 4.55]^ab^  Greater frequency of CA inc. odds of suicide ideation, aOR = 1.09 [1.04, 1.13]^ab^  Greater frequency of CA inc. odds of suicide ideation, aOR = 1.07 [1.02, 1.12]^abc^  CEA inc. odds of suicide ideation, aOR = 4.74 [2.24, 10.04]^a^  CEA inc. odds of suicide ideation, aOR = 2.78 [1.22, 6.30]^ab^  CEA inc. odds of suicide ideation, aOR = 4.20 [1.36, 13.02]^ac^  CPA inc. odds of suicide ideation, aOR = 2.80 [1.32, 5.91]^a^  CPA inc. odds of suicide ideation, aOR = 2.83 [1.29, 6.19]^ab^  CPA inc. odds of suicide ideation, aOR = 2.99 [1.23, 7.98]^ac^  CPA inc. odds of suicide ideation, aOR = 3.63 [1.32, 10.03]^abc^  CSA inc. odds of suicide ideation, aOR = 2.54 [1.10, 5.89]^a^  CSA inc. odds of suicide ideation, aOR = 2.48 [1.00, 6.12]^a b^  Any 2 CA inc. odds of suicide ideation, aOR = 4.91 [1.37, 17.65]^a^  CA & no antenatal dep inc. odds of suicide ideation, aOR = 2.57 [1.17, 5.67]^a^‡  No CA & antenatal dep inc. odds of suicide ideation, aOR = 6.72 [3.89, 11.61]^a^‡  CA & antenatal dep inc. odds of suicide ideation, aOR = 17.78 [7.20, 43.92]^a^‡ | NA, ‡ & dep | |
| Zhang (2021) | Subjective memory impairment | a = age, education, and parity  b = other abuses | Multivariate Lin. Reg. | **Prospective memory**  CEA predicts prospective memory impairment, β = 0.17^a^***  CPA predicts prospective memory impairment, β = 0.05^a^*  CSA predicts prospective memory impairment, β = 0.07 ^a^**  CEA (only) predicts prospective memory impairment, β = 0.06^ab^*  Both CEA & CPA predicts prospective memory impairment, β = 0.06^ab^*  CEA, CPA & CSA predicts prospective memory impairment, β = 0.06 ^ab^*  **Retrospective memory**  CEA predicts retrospective memory impairment, β = 0.17^a^***  CPA predicts retrospective memory impairment, β = 0.05^a^**  CSA predicts retrospective memory impairment, β = 0.08^a^***  CEA (only) predicts retrospective memory impairment, β = 0.05^ab^*  Both CEA & CSA predict retrospective memory impairment, β = 0.05 ^ab^*  Both CEA & CPA predict retrospective memory impairment, β = 0.07 ^ab^**  CEA, CPA & CSA predict retrospective memory impairment, β = 0.05 ^ab^* | NA | |
| Zhong (2016) | Suicide ideation | a = age, maternal race/ethnicity, b = IPV exp, c= dep status | Log. Reg. | Any CPA or CSA inc. odds of suicidal ideation early preg, aOR = 3.85 [2.84, 5.23]^a^  Any CPA or CSA inc. odds of suicidal ideation early preg, aOR, 2.90 [2.12, 3.97]^abc^  CPA (only) inc. odds of suicidal ideation, aOR = 2.30 [1.64, 3.22]^abc^  CSA (only) inc. odds of suicidal ideation, aOR = 2.58 [1.62, 4.09] ^abc^  CPA/CSA inc. odds of suicidal ideation, aOR = 4.04 [2.88, 5.68] ^abc^  ≥6 CA events inc. odds of suicidal ideation, aOR = 5.30 [3.36, 8.37] ^abc^  **Stratified by dep status**  The odds of suicidal ideation inc. among women with a history of any CA, irrespective of dep status: No dep, aOR = 2.73 [1.90, 3.92] ^ab^; Dep, aOR = 3.44 [1.84, 6.43] ^ab^ | NA | |

*Note.* ACES – Adverse Childhood Experiences, aOR = adjusted odds ratio, aPR = adjusted prevalence ratio, aRR = adjusted risk ratio, BMI = body mass index, BV = bacterial vaginosis, b/w = between, CA = child abuse, CAR = Cortisol Awakening Response, CEA = childhood emotional abuse, CMD = Common Mental Disorders, CPA = childhood physical abuse, CPY = childhood psychological abuse, CS = Cesarean section, CSA = childhood sexual abuse, def = definition, demos = demographics, dep = depression, discrim = discrimination, ED = eating disorder, exp = exposure, FoC = Fear of childbirth, GDM = Gestational diabetes mellitus, gest = gestation, GLM = generalized linear models, inc. = increases, HLR = hierarchical linear regression, inc = increases, IPV = intimate partner violence, Lin. Reg. = linear regression, LIPV = Lifetime IPV, Log. Reg. = logistic regression, mths = months, Multiple Reg. = Multiple linear regression, NA = no abuse, NLR = Neutrophil-lymphocyte ratio, n/s = not stated, OR = odds ratio, PA = placental abruption, phy = physical, PP = postpartum, PND = postnatal depression, PPD = postpartum depression, PrA = pregnancy-related anxiety, preg = pregnancy, prev = previous, PTB = preterm birth, PTSD = post-traumatic Stress Disorder, ref = reference, REML = Restricted Maximum Likelihood Method, RR = risk ratio, r’ship = relationship, SD = standard deviation, Reg = regression, SEM = structural equation modelling, SES = socioeconomic status, US = united states, wks = weeks, yr = year, Significance = * *p* < .05, ** *p* < .01, *** *p* < .001, **** *p* < .0001.
