# Supplementary material for: Childhood abuse and perinatal outcomes for mother and child: A systematic review of the literature
Source: PLoS One. 2024 May 24;19(5):e0302354. doi: 10.1371/journal.pone.0302354 (PMC11125509; doi:10.1371/journal.pone.0302354)
Supplement: Attachment — Submitted filename: Response to reviewers.docx [file pone.0302354.s006.docx]

Dear Editor,

Thank you for the opportunity to revise this manuscript. I also wish to thank the reviewers as their feedback was most helpful. Below, I have addressed each point and shown where I have revised the manuscript. Please do not hesitate to seek further clarification if needed.

Kind regards

Robyn

**Please include captions for your Supporting Information files at the end of your manuscript, and update any in-text citations to match accordingly. Please see our Supporting Information guidelines for more information:** [**http://journals.plos.org/plosone/s/supporting-information**](http://journals.plos.org/plosone/s/supporting-information)**.**

*This has been done.*

**Please review your reference list to ensure that it is complete and correct. If you have cited papers that have been retracted, please include the rationale for doing so in the manuscript text, or remove these references and replace them with relevant current references. Any changes to the reference list should be mentioned in the rebuttal letter that accompanies your revised manuscript. If you need to cite a retracted article, indicate the article’s retracted status in the References list and also include a citation and full reference for the retraction notice.**

*The reference list is relatively unchanged with only one new inclusion (i.e., Aslan Çetİn et al.)*

**Additional Editor Comments:**

Dear authors, minor revisions were requested by one of the reviewer and approved by this academic editor. Please make these minor revisions and submit the revised version as soon as possible.


[Note: HTML markup is below. Please do not edit.]

**Reviewers' comments:**

Reviewer's Responses to Questions

**Comments to the Author**

1. Is the manuscript technically sound, and do the data support the conclusions?

The manuscript must describe a technically sound piece of scientific research with data that supports the conclusions. Experiments must have been conducted rigorously, with appropriate controls, replication, and sample sizes. The conclusions must be drawn appropriately based on the data presented.

Reviewer #1: Yes

2. Has the statistical analysis been performed appropriately and rigorously?

Reviewer #1: N/A

3. Have the authors made all data underlying the findings in their manuscript fully available?

The [PLOS Data policy](https://aus01.safelinks.protection.outlook.com/?url=http%3A%2F%2Fwww.plosone.org%2Fstatic%2Fpolicies.action%23sharing&data=05%7C02%7Crbrunton%40csu.edu.au%7C473ba582720349cca06f08dc0dc36986%7Cf0f76207a6104fc0b4a35d797fe5283c%7C0%7C0%7C638400380332842066%7CUnknown%7CTWFpbGZsb3d8eyJWIjoiMC4wLjAwMDAiLCJQIjoiV2luMzIiLCJBTiI6Ik1haWwiLCJXVCI6Mn0%3D%7C3000%7C%7C%7C&sdata=t7O2N4tW26SqgZ0YjXMPLyva0oSJYVe7aC2UB7Mmg%2FE%3D&reserved=0) requires authors to make all data underlying the findings described in their manuscript fully available without restriction, with rare exception (please refer to the Data Availability Statement in the manuscript PDF file). The data should be provided as part of the manuscript or its supporting information, or deposited to a public repository. For example, in addition to summary statistics, the data points behind means, medians and variance measures should be available. If there are restrictions on publicly sharing data—e.g. participant privacy or use of data from a third party—those must be specified.

Reviewer #1: Yes

4. Is the manuscript presented in an intelligible fashion and written in standard English?

PLOS ONE does not copyedit accepted manuscripts, so the language in submitted articles must be clear, correct, and unambiguous. Any typographical or grammatical errors should be corrected at revision, so please note any specific errors here.

Reviewer #1: Yes

**5. Review Comments to the Author**

**Reviewer #1: This is very well written and an incredibly thorough review of the literature regarding child maltreatment and perinatal health and outcomes. If anything, there is too much information included in it; as a reader, I felt overwhelmed by the sheer volume of data being conveyed, and it read as if the authors were including absolutely every possible finding from the review, whether it made theoretical sense or was of clinical importance or not (e.g., is there tremendous value in a single study finding that CSA was associated with not attending birthing classes or doing so w/o a partner?).

If the author feels strongly about reporting all of the findings, I wonder if the they could consider dividing the contents into separate papers (e.g., ante/postpartum outcomes or type of outcome).

A second possibility would be for the author to begin each of the separate topical paragraphs with a summary of what they found, and then use SOME of the data as illustrations. I am not convinced that include ALL of the details that are included are necessary; at times these details became cumbersome to follow without re-reading multiple times, especially when the authors were describing multiple studies in the same paragraph. The minutia is really overwhelming. One example is on pg 22, where the author writes "In studies that examined all abuse types, CPA, CSA, and CEA independently predicted PPD; however, only CPA remained significant when controlling for other abuses (30). CEA and CSA were also risk factors for early-onset PPD, and CEA, CPA, and CSA were all risk factors for late-onset. The risk remained for CSA and early-onset PPD and CEA and late-onset PPD after adjusting for sociodemographic factors and other abuse types." Frankly, I got hopelessly lost by the end of the paragraph about what I was supposed to take away.**

*We appreciate the reviewer's assessment and concur that the reporting of the results is very* *detailed. We have therefore included a brief summary for each topical area, as suggested, when there are multiple studies reported. The summaries are in italics to differentiate them from the main findings. We have stated this in the paper directly below “Findings from studies” as below:*

“For each subsection of the four main categories of findings, the main findings are summarized (in italics) when multiple studies are reported. Then, the more detailed findings are provided.”

*Also, we have included additional subheadings and revised the text to provide further clarity. We understand that some findings may seem inconsequential to some readers, but we feel that this paper will have broad appeal to clinicians, researchers and academics and thus feel it important to include all studies that were identified. However, if the editor feels that further reduction is needed (i.e. report findings for illustrative purposes) please let me know.*

**If the author chooses to keep the paper in the long form that it is, please provide a description at the start of the results section that clearly illustrates how the results will be organized, and make those breaks more evident in the paper itself.**

*See above.*

**Circling back to methods, why were adolescents excluded?**

*We were interested in adult outcomes only. Adolescent pregnancy has unique challenges and we have added a brief justification to the paper for this. We have also changed “teen” to adolescence in the paper.*

“We excluded adolescent samples, as they have unique psychosocial and biological challenges (12) however papers that included some adolescents in their sample (e.g., Ranchod et al., 2016) were included.”

**Did you exclude any paper that included both adolescents and adult women?**

*No, we only excluded studies that examined adolescents. This has been clarified, see above.*

**When the author says (pg 6) that "remaining studies were individually assessed due to budget limitations" does this means that of the 90+ articles, 80 were reviewed by one individual? This seems like it could represent a limitation, even if there was good reliability in the 10 articles that were reviewed with the research assistant.**

*Yes that is correct. This relates to the quality assessment only and has been added as a limitation.*

“Also, the quality assessment was mostly conducted by one rater (due to budgetary constraints) which is a limitation that should be noted.”

**Toward the end of the paper, the authors used "cf." to denote the comparison group; either do this throughout the paper or do not. I found it useful, frankly.**

*We have used this abbreviation only when using brackets. Have added additional to the paper and revised all those in brackets for consistency.*

**At least twice, the author included information about racial stratification, but it was not clear how this provided useful insight. For example, on Pg 10, they describe a study comparing various types of abused and non-abused women and the outcome of persistent bacterial vaginosis, noting that the association was stronger for Black people compared to non-Black people; is there any theoretical or biological reason why race should impact this association? If not, why report this. If so, say why this matters.**

*We have added (at the first mention of race) that it is a known risk factor for various adverse outcomes, to clarify the reason for reporting this.*

“Moreover, when stratified by race (a known risk factor for various adverse perinatal outcomes), the magnitude of this association was stronger for non-black people than black people.”

**I appreciate the conclusion that all pregnancy-related care should be trauma-informed, and that clinicians need to be aware that childhood experiences with abuse are likely to impact ante- and postnatal health and wellbeing (just as recent trauma is). I also appreciate the critique of current approaches to studying child abuse (e.g., the design of studies needs to be tailored to the population of interest).**

*Thank you for this positive feedback.*

**Could the author be more clear in the paragraph on pg 32 about the prevalence of different types of abuse being more common in different parts of the world? If different forms of child abuse are not studied as substantially in some parts of the globe (e.g., Africa, South America), then how can there be any certainty that rates are actually higher or lower in these regions? Stigma and discomfort discussing abuse (especially sexual abuse) may be at play, and without asking questions that are specifically intended to measure experiences of abuse in these communities and cultures, prevalence estimates will not be accurate.**

*We have added additional below (new addition highlighted) to clarify.*

While this disparity may relate to differing conceptualisations of abuse (e.g., [punitive parenting practices may be acceptable in some cultures and not others), or differing methodologies for assessing abuse (125) they also highlight the disparity between the countries where child abuse is predominantly studied and the absence of studies in countries with a higher abuse prevalence (e.g., Africa and South America).

**I appreciate that in the discussion, the author did summarize the more common findings and occasionally proposed some possible reasons for WHY child abuse may be related to these outcomes. However, some of these pathways are very complex and related to one another, yet the author treats each as if they are siloed (e.g., child abuse and IPV are related; child abuse and depression are related; it seems likely that these factors may influence one another in ways that are difficult for these types of quant studies to get at). This is a limitation of looking at a subject as complex as this is in this way.**

*The reviewer makes an important point and we have added the following to the Discussion section, under Methodological issues and implications for research*

“Also, it is important to acknowledge that some of the pathways between child abuse and the outcomes reported are complex. However, few studies identified in this review examined mediation/moderation or interact effects, focussing instead on the direct relationship however the influence of other variables needs to be considered when examining the sequalae of child abuse. “

**In conclusion, this systematic review appears to be very thorough, well done, and well written. But it is easy to lose the forest for the trees with the amount of detail included. Not all of the associations that were identified from the high-quality studies seem to have clinical importance, however, and the amount of detail included often left me unable to discern what WAS important to take away.**

*I want to thank the reviewer for their insightful and helpful comments and trust that the revisions have addressed their concerns thoroughly. If, however, they feel I have not addressed them, please let me know.*
